# Screening of serum biomarkers for coronary artery calcification using DIA quantitative proteomics and construction of a regression model

**DOI:** 10.3389/fcvm.2026.1824102

**Published:** 2026-06-01

**Authors:** Ruyan Cui, Xiaoyu Liu

**Affiliations:** Department of Cardiology, Affiliated Hospital of Inner Mongolia Medical University, Hohhot, China

**Keywords:** coronary artery calcification, proteomics, data-independent acquisition, SMOC1, HSP90B1, OPTN, scatter plot, Biomarker

## Abstract

**Background:**

Using DIA quantitative proteomics technology to screen for coronary artery calcification (CAC)-associated differentially expressed proteins, this study explores the value of SMOC1, HSP90B1, and OPTN as potential serum biomarkers for CAC and constructs an individualized risk prediction model.

**Methods:**

A two-stage case-control design was employed, enrolling 320 patients divided into a CAC group (160 cases) and a non-calcified control group (160 cases). Sixty cases were stratified and randomly selected for the discovery cohort (30 cases each), with the remaining 260 cases forming the validation cohort. The discovery cohort samples were randomly divided into three biologically replicated subgroups. Serum samples were pooled in equal volumes for DIA proteomics analysis to screen differentially expressed proteins, followed by bioinformatics analysis. Candidate proteins in the validation cohort were measured via ELISA. Single-factor and multivariate logistic regression analyses were conducted using clinical data to construct a nomogram prediction model. Model performance was evaluated using ROC curves, calibration curves, and decision curves.

**Results:**

A total of 39 differentially expressed proteins were identified (18 upregulated and 21 downregulated). GO enrichment analysis revealed that the differentially expressed proteins were enriched in protein folding, immune receptor signaling pathways, and apoptosis process regulation. KEGG enrichment analysis showed enrichment in T cell receptor signaling pathways, Notch signaling pathways, and calcium reabsorption pathways. Based on the significance of differential expression, biological relevance, and literature evidence, SMOC1 (upregulated), HSP90B1 (downregulated), and OPTN (downregulated) were identified as candidate proteins. ELISA results from the validation set showed that the expression trends of these three proteins were fully consistent with the proteomics findings (*P* < 0.001). Multivariate logistic regression analysis identified age, serum uric acid, alkaline phosphatase, fasting blood glucose, SMOC1, HSP90B1, and OPTN as independent predictors of CAC (*P* < 0.05). The AUC of the logistic regression model constructed using the above indicators for predicting CAC was 0.894 (95% CI: 0.855–0.933), which was significantly higher than that of the baseline model containing only clinical indicators (AUC = 0.845; DeLong test, *P* = 0.0013); The Net Rating Improvement (NRI) was 0.908 (95% CI: 0.707–1.123), and the Integrated Discrimination Improvement (IDI) was 0.128 (95% CI: 0.090–0.167). Calibration curves demonstrated good agreement between predicted and observed probabilities, while decision curves indicated positive clinical net benefit within the 0.1–0.8 probability threshold range, significantly outperforming traditional clinical models.

**Conclusion:**

SMOC1, HSP90B1, and OPTN are potential serum biomarkers for coronary artery calcification (CAC). A nomogram model incorporating these three biomarkers and clinical indicators performs well in predicting CAC.

## 1 Introduction

Cardiovascular disease (CVD) ranks as the leading cause of death worldwide, with its prevention and control centered on effective intervention for coronary atherosclerotic heart disease (CHD). Coronary atherosclerosis constitutes the primary pathological basis of CHD, and coronary artery calcification (CAC) serves as its characteristic radiological biomarker. Multiple clinical studies have confirmed that CAC possesses diagnostic and prognostic predictive value surpassing traditional risk factors (1). Currently, CAC has been incorporated into mainstream international cardiovascular risk assessment systems as a key independent indicator for cardiovascular event risk stratification (2). Coronary artery calcification refers to the pathological process of ectopic calcium salt deposition in the vascular wall. Its mechanism resembles bone formation, regulated by multiple factors, constituting a highly ordered active biological process. The pathogenesis of CAC is complex, involving multiple pathways including inflammatory responses, extracellular matrix remodeling, and osteoblastic transformation of smooth muscle cells, and is influenced by genetic, metabolic, and environmental factors. However, current understanding of the molecular mechanisms and specific signaling pathways underlying CAC development remains incomplete, limiting the identification of early intervention targets and the formulation of precision treatment strategies.

Currently, CAC risk assessment primarily relies on traditional clinical indicators such as age, blood lipids, blood pressure, and blood glucose. However, conclusions from related studies remain controversial, and predictive efficacy is limited (3). Therefore, elucidating the molecular mechanisms of CAC, identifying novel biomarkers, and discovering intervention targets have become key research priorities and challenges. Proteomics reveals disease-specific protein molecules at the systems level by comparing protein expression profiles between normal and pathological states, providing crucial clues for exploring disease mechanisms, discovering biomarkers, and screening drug targets (4). Compared to genomics and transcriptomics, proteomics more directly reflects cellular functional states, offering essential complementary insights into complex pathophysiological processes. Data Independent Acquisition (DIA) technology, a major breakthrough in recent years in proteomics, combined with high-resolution mass spectrometry, enables high-throughput, high-accuracy, and highly reproducible quantitative analysis of proteins in large-scale samples. It offers significant advantages over the traditional Data Dependent Acquisition (DDA) model (5). Therefore, this study employs a two-stage design: first, screening differentially expressed proteins using proteomics techniques, followed by independent large-sample validation of their feasibility as biomarkers and the construction of clinical prediction models.

As a visual predictive tool, risk prediction models integrate the regression coefficients of multiple risk factors to quantify each factor's contribution to the outcome variable, thereby enabling intuitive and accurate predictions of individual event risk. This strategy has been widely applied in fields such as oncology and cardiovascular disease. However, to our knowledge, no previous study has integrated serum biomarkers identified through DIA quantitative proteomics with clinical indicators to construct a CAC risk prediction model, particularly one reporting SMOC1, HSP90B1, and OPTN as a combined serum biomarker panel. Therefore, this study aims to use DIA quantitative proteomics to screen for CAC-associated differentially expressed proteins, validate their feasibility as serum biomarkers using independent samples, and further integrate clinical data to construct a CAC risk prediction nomogram model. It should be noted that this study follows the standard “discovery–internal validation–external validation” paradigm for biomarker research, aiming to complete the first two phases (single-center discovery and independent internal validation); it is classified as an exploratory study. The constructed predictive model serves as a proof-of-concept, and its generalizability requires confirmation through future multicenter external studies. This study seeks to provide new insights and tools for the early identification, risk stratification, and personalized prevention and treatment of CAC.

## 2 Materials and methods

### 2.1 Study population

A total of 320 patients who visited the Department of Cardiovascular Medicine at the Affiliated Hospital of Inner Mongolia Medical University between February 2024 and December 2024 were enrolled as study subjects. All patients underwent coronary computed tomography angiography (CCTA). Coronary artery calcium score (CACS) was calculated using the Agatston scoring system. Patients with CACS > 0 were assigned to the coronary artery calcification group (CAC group, *n* = 160), while those with CACS = 0 were assigned to the non-calcification control group (non-CAC group, *n* = 160). Two cardiologists, unaware of the patients' clinical information, independently assessed calcification in the angiographic images. Cases with discrepancies were adjudicated by a third expert. All patient inclusion and exclusion criteria adhered to the following standards. CAC group inclusion criteria: ① Complete imaging and clinical data with accurate CCTA assessment; ② Age >18 years, male or non-pregnant female; ③ No history of myocardial infarction, arrhythmia, or hematologic disorders; ④ Informed consent obtained and signed. Exclusion criteria: ① Concurrent malignant tumors, infectious diseases, or immune system disorders; ② Abnormal liver or kidney function; ③ Coronary artery bypass grafting or stent implantation within the past 6 months; ④ Allergy to CT contrast agents or inability to cooperate with the examination; ⑤ Abnormal calcium or phosphorus metabolism; ⑥ Cognitive or psychiatric disorders; ⑦ Continuous use of uric acid-elevating medications within the past month; ⑧ Severe malnutrition; ⑨ Poor treatment compliance. This study was approved by the Ethics Committee of the Affiliated Hospital of Inner Mongolia Medical University (Approval No.: KY2025021), and all patients signed informed consent forms.

### 2.2 Clinical indicator data collection

#### 2.2.1 General data collection

Basic case information for subjects in both groups was recorded, including age, gender, body mass index (BMI), smoking history, alcohol consumption history, diabetes history, and hypertension history.

#### 2.2.2 Clinical indicator collection

On the morning of the second day after admission, fasting blood samples were drawn from the elbow vein of patients in both groups to measure triglycerides (TG), fasting blood glucose (FBG), high-density lipoprotein cholesterol (HDL-C), alkaline phosphatase (ALP), lipoprotein (a) [Lp(a)], uric acid (BUA), serum calcium, and serum phosphorus. Procedures were strictly followed according to the manufacturer's instructions.

#### 2.2.3 CCTA assessment

Patients underwent CT scanning. Lesions >0.5 mm^2^ with CT values > 130 HU were defined as calcifications. Calcification severity was assessed using the Agatston score, calculated as the product of the calcification density score and calcified area. Scoring criteria: 130–199 HU = 1 point, 200–299 HU = 2 points, 300–399 HU = 3 points, ≥400 HU = 4 points. Calcified area is measured in mm^2^. The scores for each coronary artery across all CT slices are summed to yield the total CACS. A CACS > 0 indicates calcified plaques, while CACS = 0 indicates non-calcified plaques.

Additional Methods: Detailed CCTA protocol and CACS assessment. All scans were performed using a SOMATOM Force dual-source CT scanner (Germany). Parameters: tube voltage 120 kVp, effective tube current 300 mAs, slice thickness 0.5 mm, reconstruction interval 0.4 mm. All images were evaluated by two independent cardiovascular imaging specialists; cases of disagreement were resolved by a third specialist.

### 2.3 Study design and sample grouping

This study employed a two-stage design comprising a discovery phase and a validation phase.

#### 2.3.1 Randomization method

The 320 subjects were stratified into CAC and non-CAC groups. Using a random number table method, 30 subjects were randomly selected from each group, totaling 60 subjects, to form the discovery cohort for serum proteomics analysis. The remaining 260 subjects (130 in the CAC group and 130 in the non-CAC group) formed the validation cohort for ELISA validation and predictive model development. Randomization was independently performed by researchers not involved in clinical assessment or subsequent experiments.

#### 2.3.2 Discovery set sample pooling strategy

Sample pooling during the discovery phase is a common strategy designed to balance detection sensitivity, cost-effectiveness, and biological reproducibility. Pooling samples reduces technical noise and inter-individual variability, allowing the mass spectrometer to focus on identifying proteins that are consistently altered by the disease state. To control inter-individual variation while ensuring biological replication, the 60 discovery set samples were processed as follows: The 30 serum samples from each group (CAC and non-CAC) were randomly divided into three subgroups (Calc-1/2/3 and NCalc-1/2/3) using the random number table method, with 10 samples per subgroup. Ten serum samples within each subgroup were mixed in equal volumes to form six pooled samples for subsequent proteomics analysis. Pooled sample strategies ensure sufficient detection depth while controlling inter-individual variability; they are commonly used sample processing methods in the discovery phase of proteomics. Subsequent validation using independent, large-scale ELISA assays can compensate for the loss of information at the individual level.

### 2.4 Sample processing

#### 2.4.1 Protein extraction

Take 50 μL of each sample for low-abundance protein enrichment using the magnetic bead method. Add 20 μL of magnetic beads (pre-washed once with equilibration buffer) to plasma/serum diluted with an appropriate volume of equilibration buffer. Mix thoroughly, then incubate at 37 °C on an incubator shaker at 1,000 rpm for 1 h. Place the tube on a magnetic stand for at least 2 min to remove all residual plasma/serum (taking care not to aspirate the beads). Add 200 μL of wash buffer, mix well, and incubate on an incubator shaker at 37 °C for 5 min. Finally, place the tube on a magnetic stand for 2 min. serum (taking care not to aspirate the magnetic beads), then add 200 μL of wash buffer and mix. Incubate at room temperature on a shaker at 1,000 rpm for 5 min. Finally, place on a magnetic stand for at least 2 min to remove all liquid from the tube. Repeat this washing procedure twice, for a total of three washes. Lysis and Reduction-Alkylation: Add 50 μL lysis buffer, heat at 95 °C for 10 min at 1,000 rpm on a vortex mixer, then cool to room temperature after reaction completion. Enzymatic Digestion: Add 10 μL of Trypsin and Lys-C mixed enzyme working solution. Incubate at 37 °C, 1,000 rpm on a vortex mixer for 3 h. After digestion, add 5 μL of stop solution to the tube and vortex to mix. Place on a magnetic stirrer for at least 2 min, then transfer all enzyme-treated solution to a new EP tube. Desalting and lyophilization: Desalt the enzyme-treated solution using a C18-packed tip column. Lyophilize all peptide fractions using a vacuum freeze concentrator. Finally, resuspend all peptide fractions in 0.1% FA aqueous solution. Determine peptide concentration using the BeyoBCA Peptide Concentration Assay Kit via the colorimetric method. Adjust final peptide concentration to 100 ng/μL with 0.1% FA aqueous solution.

#### 2.4.2 LC-MS/MS detection

##### 2.4.2.1 Nanoliter liquid chromatography detection

Samples were separated using the Vanquish Neo UHPLC nanoliter liquid chromatography system. Mobile phase A consisted of 0.1% formic acid aqueous solution, while phase B comprised 0.1% formic acid acetonitrile solution (acetonitrile at 100%). The injection mode employed a trap-and-run dual-column method, featuring a PepMap Neo Trap Cartridge (300 μm × 5 mm, 5 μm) as the trap column and an Easy-Spray™ PepMap™ Neo UHPLC column (150 μm × 15 cm, 2 μm) as the analysis column. The analysis column temperature was maintained at 55 °C by an integrated column oven. The injection volume was 200 ng, the flow rate was 2.5 μL/min, the effective gradient duration was 13 min, and the total run time was 14 min.

##### 2.4.2.2 Orbitrap astral mass spectrometer detection

DIA analysis employed chromatographic separation using a nanoliter-scale Vanquish Neo system (Thermo Fisher Scientific). Samples separated by nanoliter-scale HPLC underwent DIA (data-independent acquisition) mass spectrometry analysis using an Orbitrap Astral high-resolution mass spectrometer (Thermo Fisher Scientific). Detection Mode: Positive ions, parent ion scan range 380–980 m/z, MS1 resolution 240,000 at 200 m/z, Normalized AGC Target 500%, Maximum IT 5 ms. MS2 was acquired in DIA mode with a 299-mass window, 2 ps isolation window, 25% HCD collision energy, 500% normalized AGC target, and 3 ms maximum injection time.

#### 2.4.3 Mass spectrometry data analysis

The DIA mass spectrometry data in this project utilized the DIA-NN (v.1.8.1) software for library search, employing a library-free approach. Search parameters: Database: uniprotkb_proteome_UP000005640_human_83385_20250224. fasta (83,385 sequences total). Checked the deep learning-based parameter to predict a spectral library. MBR (Match Between Runs) was selected to generate a spectral library from DIA data, which was then reanalyzed to obtain protein qualitative and quantitative results. Both precursor ions and protein-level identification were filtered at an FDR (False Discovery Rate) of 1%. The filtered data were ready for subsequent bioinformatics analysis.

### 2.5 Functional analysis of differentially expressed proteins

Functional annotation and enrichment analysis of selected differentially expressed proteins were performed using Gene Ontology (GO) and the Kyoto Encyclopedia of Genes and Genomes (KEGG). GO analysis describes protein functions across three levels: Biological Process (BP), Cellular Component (CC), and Molecular Function (MF). KEGG analysis was employed to identify significantly enriched signaling pathways among the differentially expressed proteins. Enrichment analysis utilized the hypergeometric test with a significance threshold of *P* < 0.05.

### 2.6 ELISA validation

In the validation cohort, levels of human secretory modular calcium-binding protein 1 (SMOC1), human heat shock protein 90B1 (Hsp90B1), and human optic pathway protein (OPTN) in serum were detected using enzyme-linked immunosorbent assay (ELISA). All procedures were strictly followed according to the kit instructions.

### 2.7 Statistical analysis

Statistical analysis was performed using SPSS 27.0 and R software (version 4.2.0).

#### 2.7.1 Data description and comparison

First, quantitative data underwent normality testing (Shapiro–Wilk test) and homogeneity of variance testing (Levene's test). Normally distributed quantitative data were expressed as mean ± standard deviation (x ± s), and comparisons between groups were performed using the independent samples *t*-test. Non-normally distributed continuous data were expressed as median (interquartile range) [M (P_25_, P_75_)], and comparisons between groups were performed using the Mann–Whitney *U*-test. Categorical data were expressed as numbers (percentage) [*n* (%)], and comparisons between groups were performed using the chi-square test or Fisher's exact test.

#### 2.7.2 Proteomics data processing

DIA-NN software was employed for protein qualitative and quantitative analysis of raw mass spectrometry data. The UniProt human database was used for database searches, with protein identification filtered at a 1% false discovery rate (FDR). To ensure data quality, post-search quality control evaluations were performed, including peptide length distribution, peptide number distribution, missed cleavage site distribution, inter-sample abundance distribution, principal component analysis (PCA), and inter-sample correlation analysis. Protein quantitative values were normalized by median to eliminate experimental batch effects. Differentially expressed proteins were screened based on the criteria: fold change (FC) ≥1.5 or ≤0.6667, and *P* < 0.05 in *t*-tests. We applied Benjamini-Hochberg false discovery rate (FDR) correction.

#### 2.7.3 Risk factor analysis and predictive model development

Binary logistic regression analysis (using *P* < 0.05 in univariate analysis as the threshold for candidate variables in multivariate analysis) was employed to identify independent risk factors for coronary artery calcification. Odds ratios (OR) and 95% confidence intervals (CI) were calculated. Based on the logistic regression results, a regression model was constructed using the rms package in R software. To quantify the incremental predictive value of the three protein biomarkers, we calculated the Net Rating Improvement (NRI) and the Integrated Discrimination Improvement (IDI) to compare the predictive performance of the baseline model (containing only clinical indicators) with that of the full model (clinical indicators plus the three proteins). Additionally, we used the DeLong test to compare differences in AUC between the two models. Furthermore, the variance inflation factor (VIF) was employed to assess multicollinearity among predictors; a VIF less than 5 was considered indicative of no significant multicollinearity. Model discrimination was assessed using receiver operating characteristic (ROC) curves, calculating area under the curve (AUC) and 95% CI. Model calibration was evaluated by plotting calibration curves using Bootstrap resampling (B = 1,000) and combining with the Hosmer-Lemeshow goodness-of-fit test. The rmda package was used to generate decision curves (DCAs) to assess the clinical net benefit of the model at different threshold probabilities. Higher risk scores indicate a greater risk of developing CAC. All tests were two-sided, with *P* < 0.05 considered statistically significant.

## 3 Results

### 3.1 Comparison of baseline data between discovery and validation cohorts

To assess the representativeness of the discovery cohort samples for the overall study population, this study compared baseline clinical data between the discovery cohort (60 cases) and the validation cohort (260 cases). Results showed no statistically significant differences between the two groups in age, gender, BMI, smoking history, drinking history, hypertension history, diabetes history, serum uric acid, ALP, FBG, serum calcium, and serum phosphorus (*P* > 0.05), indicating high homogeneity and comparability between the two groups (Table 1). This indicates that the discovery cohort obtained through stratified random sampling effectively represents the overall study population, establishing a reliable foundation for subsequent validation in the validation cohort.

**Table 1:** Comparison of baseline data between discovery and validation sets.

Variable	Discovery Collection (*n* = 60)	Validation set (*n* = 260)	t/Z	P
Diabetes (*n*, %)	12 (20.0)	53 (20.4)	–	1.000
Drink (*n*, %)	12 (20.0)	60 (23.1)	–	0.732
Hypertension (*n*, %)	32 (53.3)	144 (55.4)	–	0.776
Male (*n*, %)	26 (43.3)	95 (36.5)	–	0.376
Smoke (*n*, %)	25 (41.7)	123 (47.3)	–	0.474
ALP[U/L, M(P25, P75)]	72.00 (65.00, 83.00)	72.00 (64.00, 83.00)	−0.17	0.865
BMI[kg/m^2^, M(P25, P75)]	20.65 (19.30, 24.00)	20.70 (19.23, 23.48)	0.582	0.561
FBG[mmol/L, M(P25, P75)]	4.90 (4.46, 5.68)	5.09 (4.50, 5.98)	−0.917	0.359
HDL[mmol/L, M(P25, P75)]	1.16 (0.96, 1.34)	1.15 (0.93, 1.37)	0.427	0.670
LDL[mmol/L, M(P25, P75)]	2.19 (1.84, 2.63)	2.21 (1.76, 2.90)	−0.44	0.661
Lp(a)[g/L, M(P25, P75)]	0.17 (0.09, 0.32)	0.14 (0.07, 0.29)	1.098	0.272
P[mmol/L, M(P25, P75)]	1.06 (0.92, 1.18)	1.02 (0.92, 1.13)	0.754	0.451
TC[mmol/L, M(P25, P75)]	3.99 (3.46, 4.75)	3.96 (3.47, 4.85)	−0.161	0.873
TG[mmol/L, M(P25, P75)]	1.21 (0.83, 1.76)	1.30 (0.96, 1.85)	−1.033	0.302
Age[years, M(P25, P75)]	65.00 (64.00, 68.25)	65.00 (63.00, 69.00)	−0.009	0.993
BUA (μmol/L, $\bar{x}\pm s$)	320.40 ± 47.02	311.74 ± 49.08	1.241	0.215
Ca ((mmol/L, $\bar{x}\pm s$)	2.19 ± 0.12	2.22 ± 0.13	−1.716	0.087

Note: For normally distributed quantitative data, results are expressed as mean ± standard deviation (x ± s); for non-normally distributed data, median (interquartile range) [M (P25, P75)] is used. Count data are presented as number (percentage) [n (%)]. Intergroup comparisons employed independent samples *t*-tests (for normally distributed quantitative data), Mann–Whitney *U*-tests (for non-normally distributed quantitative data), or *χ*^2^-tests (for categorical data). When theoretical frequency <5 or total sample size <40, Fisher's exact probability test was used (indicated by “–” in tables). *P* < 0.05 was considered statistically significant. ALP, Alkaline phosphatase; BMI, Body mass index; FBG, Fasting blood glucose; HDL, High-density lipoprotein cholesterol; LDL, Low-density lipoprotein cholesterol; Lp(a), Lipoprotein(a); TC, Total cholesterol; TG, Triglycerides; BUA, Blood uric acid; Ca, Serum calcium.

### 3.2 Serum proteomics analysis of the discovery cohort

#### 3.2.1 Proteomics data quality control and global expression patterns

Based on DIA mass spectrometry data from six pooled samples in the discovery cohort, a total of 2,739 proteins were identified, with quantitative information obtained for 2,676 of them. Quality control results showed that 92.7% of peptide segments had zero missed cleavage sites, indicating thorough enzymatic digestion. Box plots and violin plots of inter-sample abundance distributions revealed highly similar patterns among biological replicates within each group, indicating excellent intra-group reproducibility (Supplementary Figure S1). Principal Component Analysis (PCA) showed that the first principal component (PC1) contributed 27.14% of the variance. Samples from the CAC group and non-CAC group exhibited clear separation in both two-dimensional and three-dimensional score plots (Supplementary Figures S2, S3). Analysis of inter-sample correlations showed that the Pearson correlation coefficients between samples within each group were all >0.95. On the one hand, this confirms the high technical reproducibility of the DIA-MS platform and rules out the interference of technical noise on the results; on the other hand, the high similarity in serum proteomic backgrounds is a typical characteristic of clinical cohort samples from the same ethnic group with similar clinical baseline characteristics. Against this backdrop, the differentially expressed proteins identified are more likely to be specific biomarkers of the CAC pathological state rather than products of individual heterogeneity (Supplementary Figure S4).

#### 3.2.2 Screening of differentially expressed proteins

**Figure 1:**
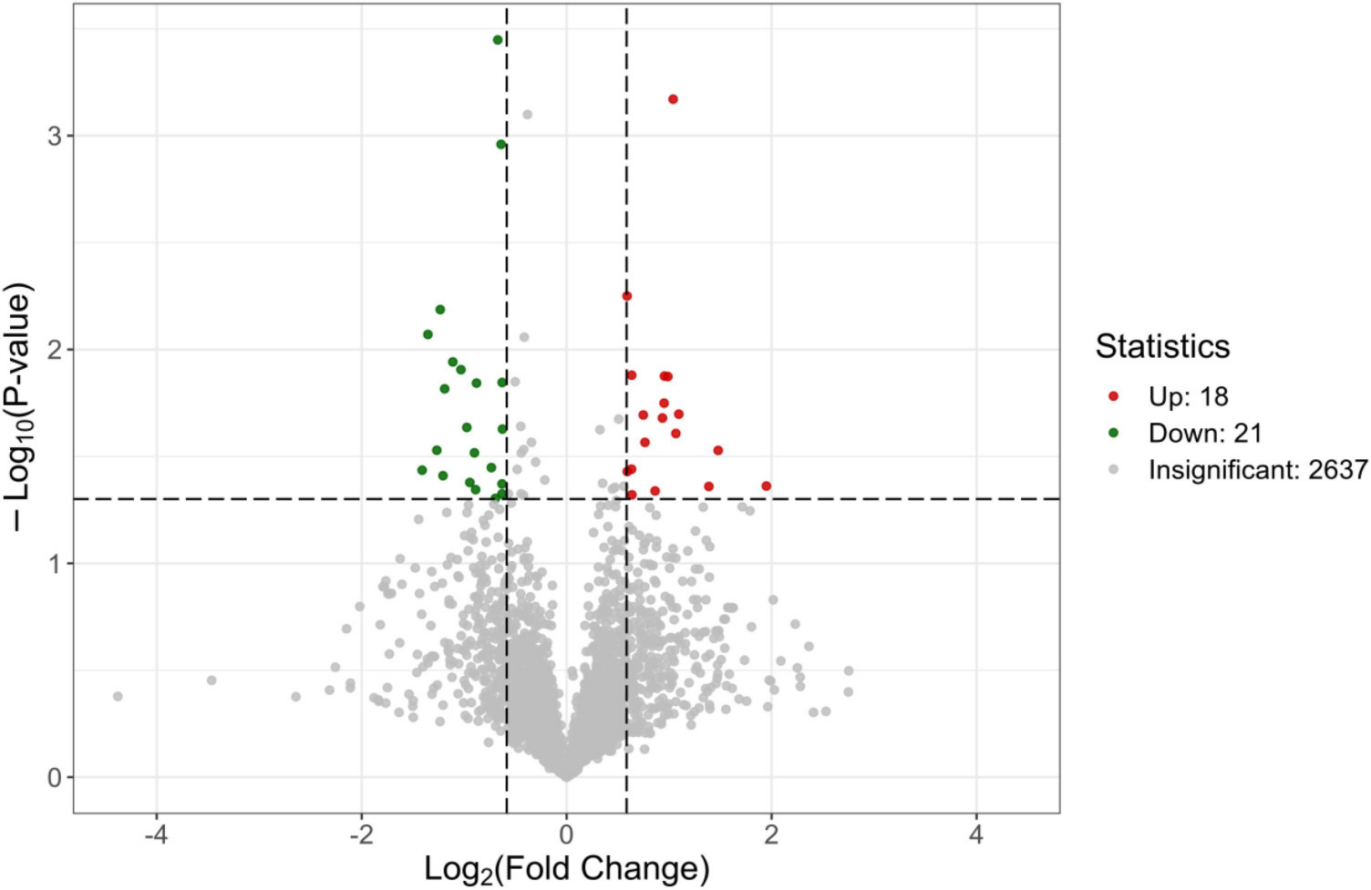
Volcano plot of differentially expressed proteins.

**Figure 2:**
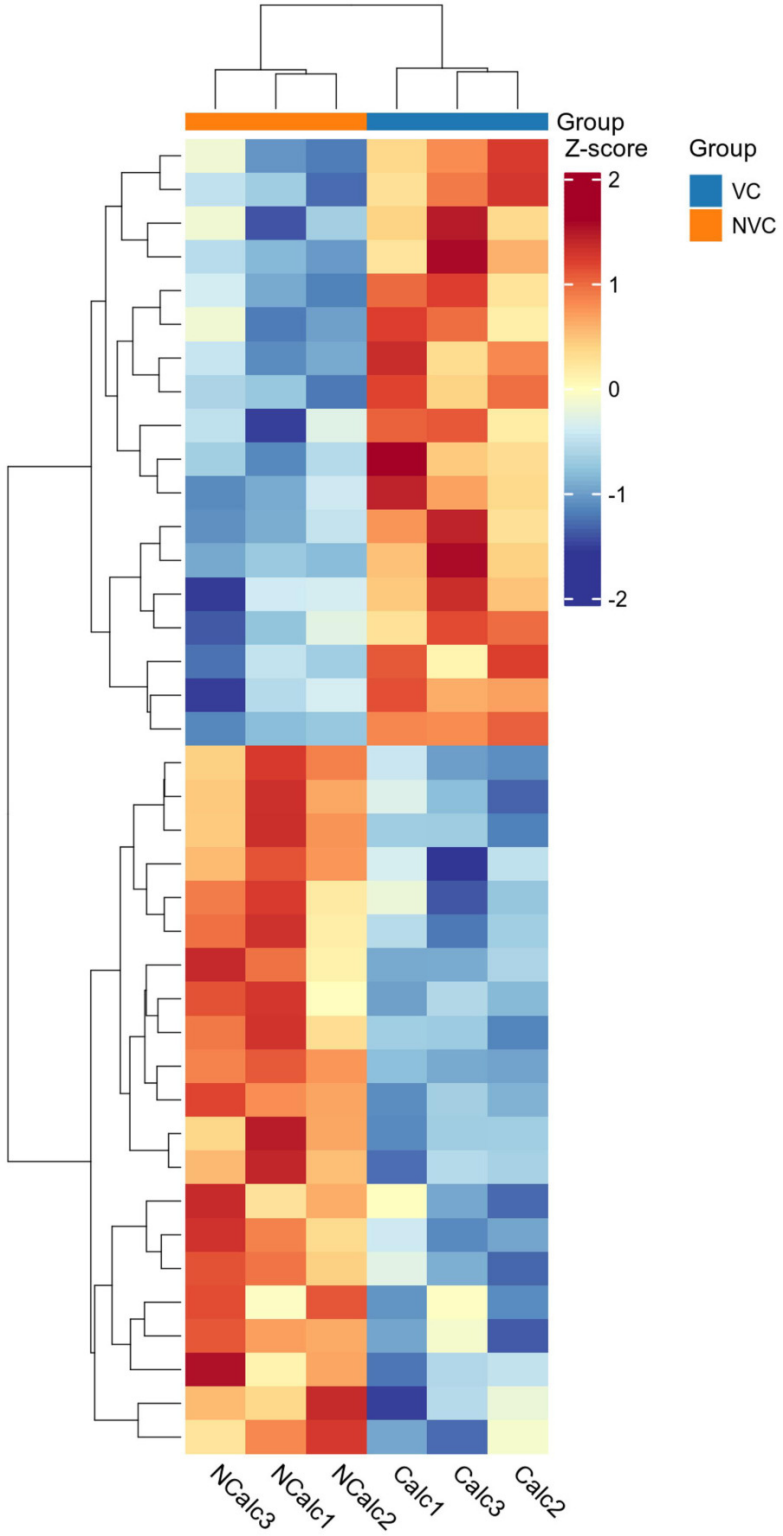
Heatmap of differentially expressed proteins clustering.

Using a fold change (FC) ≥1.5 or ≤0.6667 and *P* < 0.05 as criteria, a total of 39 differentially expressed proteins were identified, including 18 upregulated and 21 downregulated proteins in the CAC group (Table 2). A volcano plot visually displayed the distribution of differentially expressed proteins (Figure 1). Clustering heatmap analysis revealed that samples from the CAC group and non-CAC group clearly clustered into two distinct categories, with significant differences in expression patterns observed between the two groups (Figure 2).

**Table 2:** List of differentially expressed proteins between coronary artery calcification group and Non-calcification control group.

Accession	Gene	FC	*P*-value	Regulation
P17252	PRKCA	2.05489398	0.000675239	up
Q9UQ35	SRRM2	1.504920599	0.005619739	up
Q8TCD5	NT5C	1.552215864	0.013184806	up
O75487	GPC4	1.937706494	0.013316717	up
P62241	RPS8	1.982530867	0.013381255	up
Q8N2S1	LTBP4	1.932997137	0.017821308	up
P13010	XRCC5	2.134981721	0.020055773	up
D6RBQ9	HNRNPD	1.679102568	0.020238896	up
P52790	HK3	1.911492645	0.020919192	up
P31939	ATIC	2.092901586	0.024706548	up
A0A0C4DH34	IGHV4-28	1.698334089	0.027182897	up
Q9H4F8	SMOC1	2.786170463	0.029640303	up
P36578	RPL4	1.550350723	0.036282877	up
A0AAG2TF08	CANX	1.506803966	0.037224876	up
Q15833	STXBP2	3.860368808	0.043502717	up
Q9Y315	DERA	2.61691839	0.04370748	up
Q96RF0	SNX18	1.818647296	0.045863443	up
P29350	PTPN6	1.554238337	0.04781798	up
P30740	SERPIN	0.627467112	0.000356601	down
Q9NPA2	MMP25	0.641705509	0.001098015	down
Q9Y6R7	FCGBP	0.425251713	0.006504694	down
J3KSN5	USH1G	0.391267176	0.008503667	down
Q9H3G5	CPVL	0.463004698	0.01143002	down
Q9NRR1	CYTL1	0.489202925	0.012423649	down
P43251	BTD	0.64644192	0.01426334	down
Q15166	PON3	0.543418135	0.014356101	down
Q8NEJ9	NGDN	0.438083612	0.015273007	down
Q15848	ADIPOQ	0.509027766	0.0231487	down
Q13200	PSMD2	0.647639374	0.023568757	down
Q96CV9	OPTN	0.415523192	0.029592589	down
A0A8V8TNU2	WDR1	0.53589367	0.030413633	down
Q9NY33	DPP3	0.600977212	0.035671742	down
P28300	LOX	0.376211602	0.036667755	down
Q99685	MGLL	0.433260279	0.038914328	down
P42680	TEC	0.519382135	0.041853216	down
Q8NES3	LFNG	0.645894824	0.042533724	down
A6NMY6	ANXA2P2	0.54015034	0.045223555	down
Q9NVJ2	ARL8B	0.645711447	0.047331537	down
A0A7P0TAY2	HSP90B1	0.617428484	0.049683883	down

#### 3.2.3 Trend analysis of differentially expressed proteins

K-means clustering of the 39 differentially expressed proteins identified two subclasses: Subclass 1 (21 proteins) exhibited a downregulation trend in the CAC group, while Subclass 2 (18 proteins) showed an upregulation trend (Supplementary Figure S5). K-means clustering of all 2,676 quantified proteins similarly yielded two subclasses (1,321 and 1,355 proteins), exhibiting opposite overall expression trends between the two groups (Supplementary Figure S6).

### 3.3 Bioinformatics analysis of differentially expressed proteins

**Figure 3:**
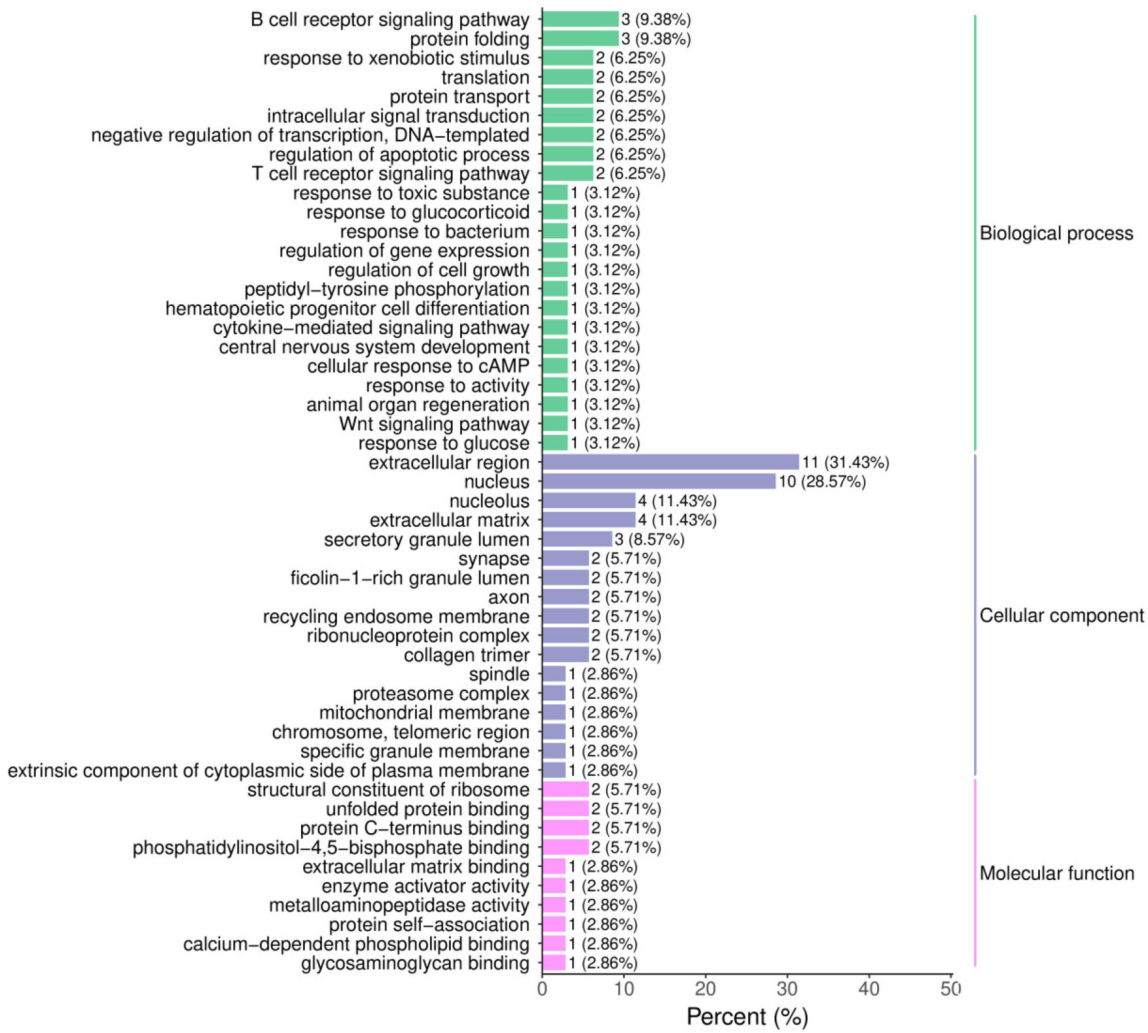
Bar chart of GO enrichment analysis for differentially expressed proteins.

**Figure 4:**
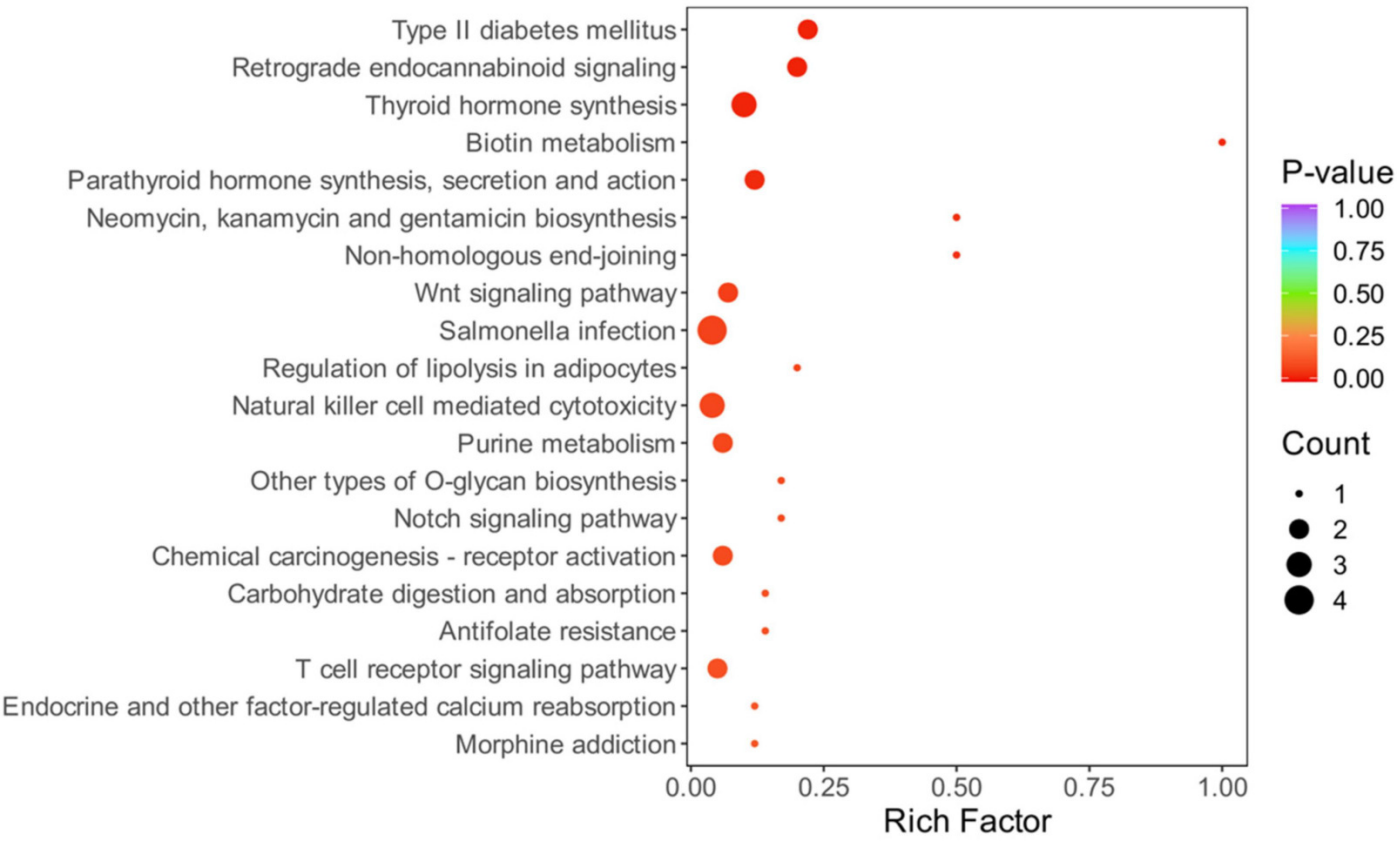
Bubble chart of KEGG pathway enrichment analysis for differentially expressed proteins.

GO enrichment analysis revealed that differentially expressed proteins were significantly enriched in multiple biological processes (BP), including protein folding, B-cell receptor signaling pathways, T-cell receptor signaling pathways, and apoptosis regulation. Cellular components (CC) were primarily localized to the extracellular region, nucleus, nucleolus, and extracellular matrix. Molecular functions (MF) were significantly enriched in phosphatidylinositol-4,5-bisphosphate binding, unfolded protein binding, ribosomal structural component, and extracellular matrix binding (*P* < 0.05, Figure 3). KEGG pathway enrichment analysis revealed that differentially expressed proteins were significantly enriched in pathways such as T cell receptor signaling, Notch signaling, and calcium reabsorption regulated by endocrine and other factors (Figure 4).

### 3.4 Screening of candidate proteins and ELISA validation

**Figure 5:**
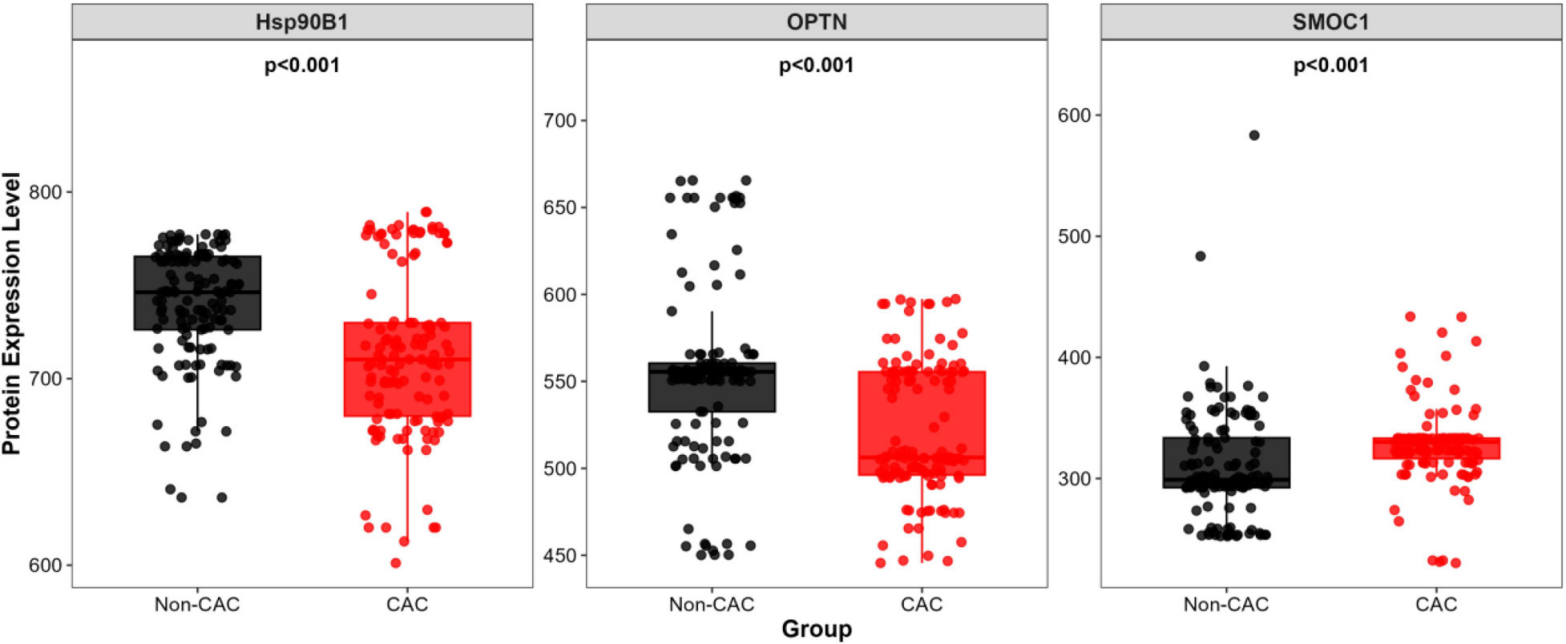
ELISA validation results for Serum levels of three candidate proteins in the validation pool.

This study further validated the candidate protein biomarkers. To ensure the objectivity and transparency of the screening process, we ranked all 39 differentially expressed proteins (DEPs) based on the following five predefined criteria: (1) Statistical significance: FC ≥ 1.5 or ≤0.6667 and *P* < 0.05 (all 39 DEPs met this criterion); (2) Biological relevance: Strong association with CAC-related pathways identified by GO/KEGG enrichment analysis (calcium binding, endoplasmic reticulum stress, autophagy, ECM remodeling); (3) Reproducibility: Consistent expression trends across three biological replicates; (4) Literature evidence: Existing functional studies supporting their roles in vascular biology, osteogenic differentiation, or calcification; (5) Assay feasibility: The availability of validated, high-quality ELISA kits for independent large-scale validation. To further assess the stability of candidate proteins during the discovery phase, we calculated the coefficient of variation (CV) for three candidate proteins (SMOC1, HSP90B1, OPTN) across three biological replicates (Supplementary Figure S7): SMOC1 had a CV of 14.69% in the non-CAC group, and 9.66% in the CAC group; HSP90B1 had a CV of 4.32% in the non-CAC group and 6.23% in the CAC group; OPTN had a CV of 8.99% in the non-CAC group and 7.31% in the CAC group. All CV values were below 15%, confirming the high stability and robustness of the three candidate biomarkers in serum samples. In the validation cohort of 260 samples, ELISA results showed (Figure 5): Serum SMOC1 levels in the CAC group were significantly higher than in the non-CAC control group: 330.13 (314.76, 333.25) ng/mL vs. 299.00 (292.43, 335.05) ng/mL, *P* < 0.001; Serum HSP90B1 levels in the CAC group were significantly lower than in the non-CAC control group: 710.26 (679.60, 730.07) ng/mL vs. 746.27 (725.51, 765.51) ng/mL, *P* < 0.001; Serum OPTN levels in the CAC group were also significantly lower than in the non-CAC control group: 506.20 (495.90, 555.46) ng/mL vs. 555.52 (530.94, 560.56) ng/mL, *P* < 0.001. The validation results were fully consistent with the trends observed in proteomics, confirming the effectiveness and unbiased nature of the screening strategy.

### 3.5 Establishment and evaluation of coronary artery calcification prediction model

#### 3.5.1 Univariate analysis of baseline characteristics between calcified and non-calcified groups

No statistically significant differences were observed between the two groups in terms of gender, smoking status, alcohol consumption, history of hypertension, history of diabetes, serum calcium, or serum phosphorus (*P* > 0.05). Statistically significant differences were observed between groups in age, blood uric acid (BUA), alkaline phosphatase, fasting blood glucose, SMOC1, Hsp90B1, and OPTN (*P* < 0.05) (Table 3).

**Table 3:** Univariate analysis of coronary artery calcification group vs. Non-calcification group.

Variable	Calcification group (*n* = 130)	Non-calcification group (*n* = 130)	*χ*2/Z	*P*
Male (*n*, %)	78 (60.00)	87 (66.90)	1.344	0.246
Smoke (*n*, %)	56 (43.10)	67 (51.50)	1.867	0.172
Drink (*n*, %)	29 (22.30)	31 (23.80)	0.087	0.768
Diabetes (*n*, %)	30 (23.10)	23 (17.70)	1.161	0.281
Hypertension (*n*, %)	76 (58.50)	68 (52.30)	0.996	0.318
Age[years, M(P25, P75)]	68 (64, 71)	64 (62, 66)	−5.330	<0.001
BMI[kg/m^2^, M(P25, P75)]	20.91 (19.19, 23.83)	20.65 (19.28, 23.30)	−1.027	0.305
LDL[mmol/L, M(P25, P75)]	2.24 (1.86, 2.88)	2.19 (1.68, 2.91)	−0.396	0.692
HDL[mmol/L, M(P25, P75)]	1.19 (0.94, 1.37)	1.11 (0.92, 1.38)	−0.738	0.460
TG[mmol/L, M(P25, P75)]	1.28 (0.96, 1.77)	1.31 (0.94, 1.98)	−0.243	0.808
TC[mmol/L, M(P25, P75)]	3.95 (3.51, 4.80)	3.98 (3.37, 4.94)	−0.120	0.905
BUA[μmol/L, M(P25, P75)]	324.00 (300.75, 355.25)	291.50 (262.75, 332.50)	−4.727	<0.001
Ca[mmol/L, M(P25, P75)]	2.22 (2.12, 2.31)	2.24 (2.13, 2.31)	−0.212	0.832
P[mmol/L, M(P25, P75)]	1.03 (0.94, 1.15)	1.00 (0.90, 1.11)	−1.543	0.123
ALP[U/L, M(P25, P75)]	78 (67.00, 86.00)	68 (61.00, 78.00)	−4.553	<0.001
Lp(a)[g/L, M(P25, P75)]	0.17 (0.09, 0.30)	0.12 (0.07, 0.26)	−1.784	0.074
FBG[mmol/L, M(P25, P75)]	5.60 (4.68, 6.87)	4.85 (4.37, 5.39)	−5.163	<0.001
SMOC1[pg/mL, M(P25, P75)]	330.13 (314.76, 333.25)	299.00 (292.43, 335.05)	−5.101	<0.001
Hsp90B1[pg/mL, M(P25, P75)]	710.26 (679.60, 730.07)	746.27 (725.51, 765.51)	−4.845	<0.001
OPTN[pg/mL, M(P25, P75)]	506.20 (495.90, 555.46)	555.52(530.94, 560.56)	−5.630	<0.001

#### 3.5.2 Multivariate logistic analysis of coronary artery calcification group vs. non-calcification group

With coronary artery calcification occurrence as the dependent variable (CAC group = 1, non-CAC group = 0), univariate analysis identified statistically significant predictors: age, serum uric acid (BUA), alkaline phosphatase (ALP), fasting blood glucose (FBG), SMOC1, Hsp90B1, and OPTN were selected as independent variables. Binary logistic regression analysis revealed that age, serum uric acid, ALP, FBG, SMOC1, Hsp90B1, and OPTN were independent risk factors for coronary artery calcification (*P* < 0.05) (Table 4). The final independent variables included in the model were age, alkaline phosphatase, fasting blood glucose, SMOC1, HSP90B1, and OPTN, all of which were independent factors influencing CAC.

**Table 4:** Multivariate logistic regression analysis of factors influencing coronary artery calcification.

Relevant factors	*β*	SE	Waldχ2	OR (95%CI)	*P*
Age	0.162	0.042	14.565	1.176 (1.082, 1.278)	<0.001
BUA	0.013	0.004	13.350	1.013 (1.006, 1.020)	<0.001
ALP	0.047	0.014	10.968	1.048 (1.019, 1.077)	<0.001
FBG	0.774	0.152	25.967	2.169 (1.610, 2.921)	<0.001
SMOC1	0.013	0.004	9.235	1.013 (1.005, 1.021)	0.002
Hsp90B1	0.017	0.004	15.760	1.018 (1.009, 1.026)	<0.001
OPTN	0.017	0.004	14.805	1.017 (1.008, 1.026)	<0.001

#### 3.5.3 Construction of the nomogram prediction model

**Figure 6:**
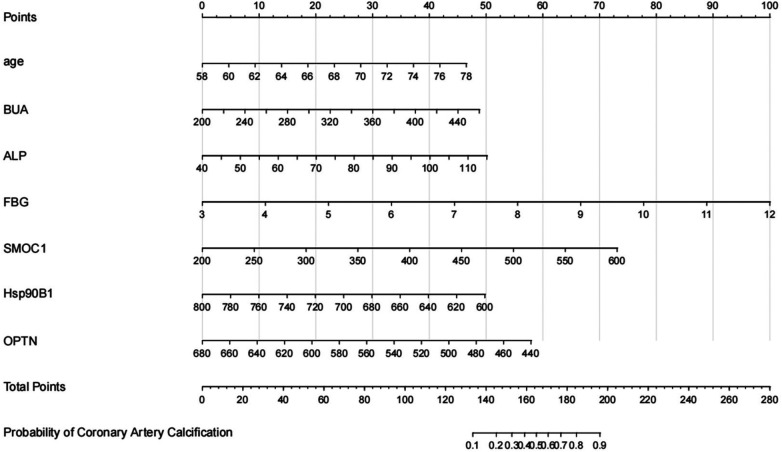
Nomogram model for predicting the risk of coronary artery calcification.

A nomogram prediction model for coronary artery calcification risk was developed based on the independent risk factors in Table 4. Results showed that for each additional year of age, the nomogram model score increased by 2.25 points; for every 40 μmol/L increase in uric acid level, the nomogram model score increased by 7.5 points; an increase of 15 U/L in ALP levels raised the score by 10 points; an increase of 1 mmol/L in FBG levels raised the score by 11.1 points; an increase of 50 pg/mL in SMOC1 levels raised the score by 9 points; A decrease of 40 pg/mL in Hsp90B1 levels resulted in a 10-point increase in the nomogram score; a decrease of 40 pg/mL in OPTN levels resulted in a 9.8-point increase in the nomogram score; (Figure 6).

#### 3.5.4 Validation and evaluation of the nomogram model

**Figure 7:**
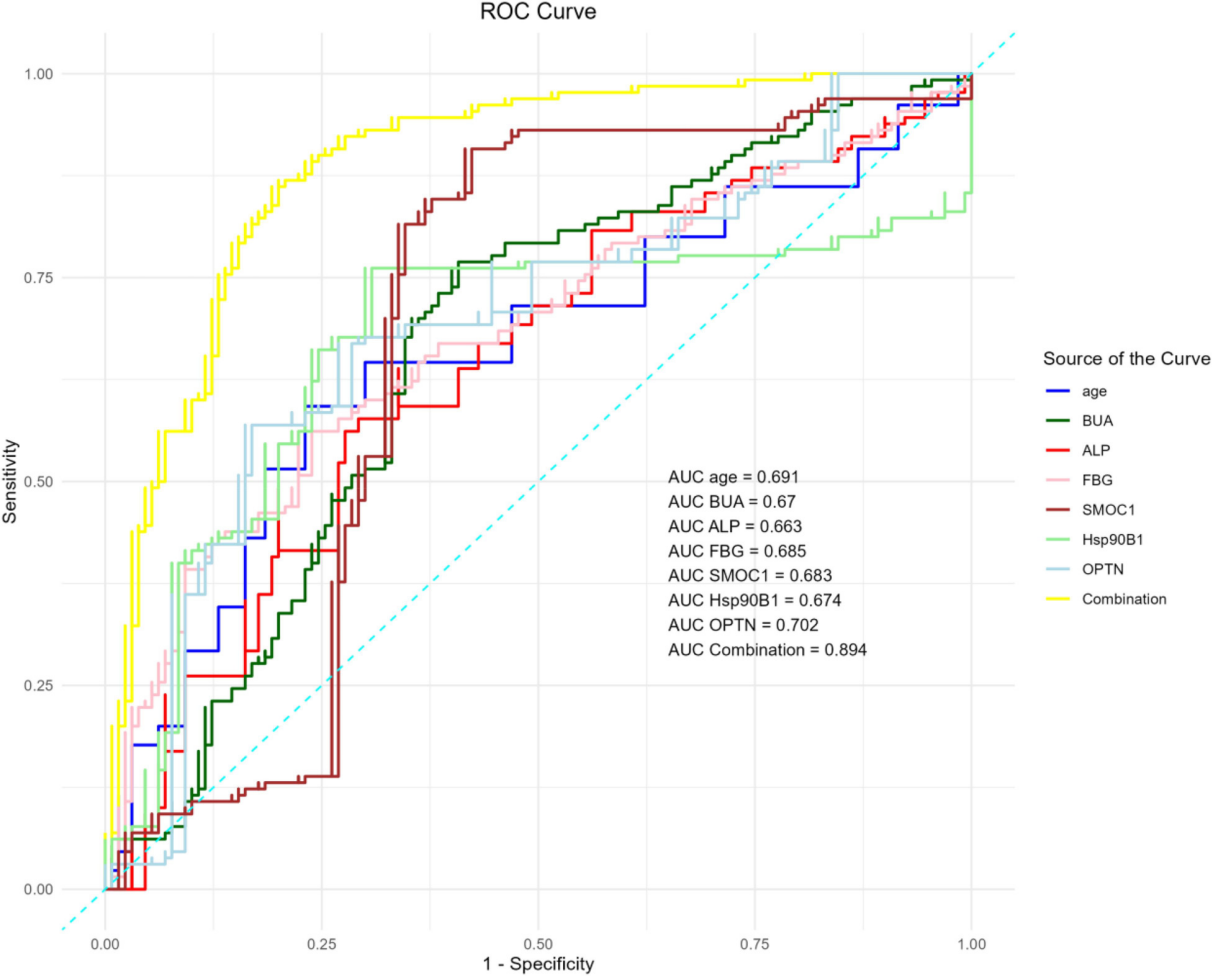
ROC curves for the regression model and Various clinical indicators in predicting coronary artery calcification.

**Figure 8:**
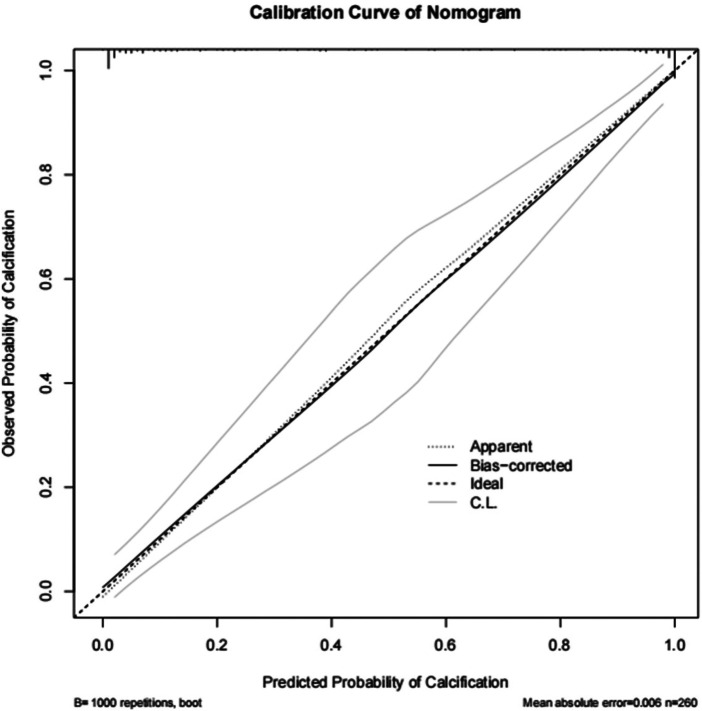
Calibration curve for the regression chart prediction model.

**Figure 9:**
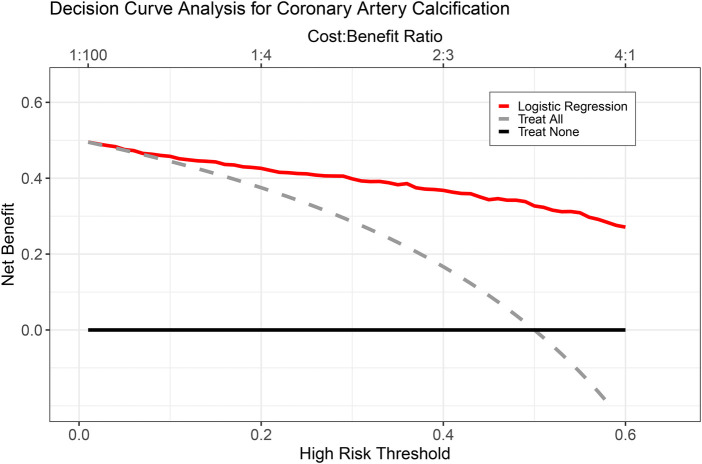
Decision curve model for regression prediction.

ROC analysis revealed that the AUC values for age, uric acid, ALP, FBG, SMOC1, Hsp90B1, and OPTN yielded AUC values of 0.691, 0.685, 0.670, 0.663, 0.683, and 0.702, respectively, with sensitivities of 0.592, 0.539, 0.762, 0.638, 0.908, 0.762, and 0.569, respectively. Their specificities were 0.769, 0.777, 0.600, 0.662, 0.585, 0.700, 0.838, respectively, indicating that all these indicators possess good discriminatory ability for coronary artery calcification (Table 5). To quantify the incremental predictive value of the three protein biomarkers, we compared the predictive performance of the baseline model (containing only age, uric acid, ALP, and FBG) with that of the full model (baseline model + SMOC1, HSP90B1, and OPTN). ROC analysis showed that the AUC of the full model was 0.894 (95% CI: 0.855–0.933), which was significantly higher than that of the baseline model (0.845; 95% CI: 0.798–0.892); ΔAUC = 0.049; DeLong test *P* = 0.0013 (Supplementary Figure S8). The Net Rating Improvement (NRI) for continuous weight classification was 0.908 (95% CI: 0.707–1.123), and the Integrated Discrimination Improvement (IDI) was 0.128 (95% CI: 0.090–0.167) (Supplementary Figure S9). Although the *P*-values for NRI and IDI were 0.521 and 0.494, respectively, and did not reach the conventional level of significance, their point estimates and lower confidence limits both indicated clear clinical improvement (the lower limit of the NRI confidence interval, 0.707, was far greater than 0, and the lower limit of the IDI, 0.090, was also >0). The probability distribution plot showed that the full model demonstrated better separation of probability distributions between the CAC and non-CAC groups compared to the baseline model (Supplementary Figure S10). We performed a multicollinearity diagnosis using the variance inflation factor (VIF) for all predictor variables in both the baseline and full models; the results are shown in Supplementary Table S1: The VIF range for variables in the baseline model was 1.033–1.115, and for the full model, it was 1.028–1.140. with all VIF values well below the conventional threshold of 5, indicating that there are no significant multicollinearity issues in the models. During the discovery phase of differentially expressed protein screening, we applied Benjamini-Hochberg FDR correction; the results are shown in Supplementary Table S2. The FDR-corrected *P*-values for all 39 differentially expressed proteins were 0.986, failing to meet the conventional significance threshold. We interpret this as follows: the discovery cohort had an extremely small sample size (only 6 pooled samples), and FDR correction tends to be overly conservative; however, all three candidate proteins exhibited highly significant differences (*P* < 0.001) in ELISA assays conducted in an independent large-sample validation cohort (*n* = 260). This independent validation result provides the strongest support for the reliability of these biomarkers. The area under the curve (AUC) for the log-line model in predicting the risk of CAC was 0.894 (95% CI: 0.855–0.894), with a Youden's index of 0.669 (threshold > 0.467), a sensitivity of 0.869, and a specificity of 0.800, indicating that the log-line model has good discriminatory power (Figure 7). The calibration curve plotted using the Bootstrap method (1,000 repeated samples) shows good agreement between the model-predicted probability of CAC occurrence and the observed probability (Figure 8), suggesting that the model is well calibrated. The decision curve analysis (DCA) (Figure 9) shows that when the threshold probability falls within a relatively wide range of approximately 0.1–0.8, the net benefit of this log-odds model is higher than that of the “all-intervention” or “no-intervention” strategies, indicating that the model has good clinical utility. “Verify the distribution of CACS in the cohort. In the non-calcified group (*n* = 130), all subjects had a CACS of 0. In the calcified group (*n* = 130), CACS exhibited a clearly right-skewed distribution (Shapiro–Wilk test, *P* < 0.001), with a mean ± standard deviation of 301.9 ± 389.5, a median [interquartile range] of 86.6 [70.6, 319.6], and a range of 49.4–1,518.9. This wide range indicates that our study population represents the full spectrum of CACS. (See Supplementary Table S3 and Figure S11)”.

**Table 5:** Clinical indicators, differentially expressed proteins, and their combined predictive value for coronary artery calcification.

Indicator	Cut-off value	Youden index	Sensitivity	Specificity	AUC	95%CI
Age (years)	66.500	0.360	0.592	0.769	0.691	0.626–0.755
FBG (mmol/L)	5.470	0.338	0.539	0.777	0.685	0.620–0.750
BUA (μmol/L)	299.500	0.362	0.762	0.600	0.670	0.603–0.736
ALP (U/L)	72.500	0.300	0.638	0.662	0.663	0.596–0.729
SMOC1 (pg/mL)	302.790	0.423	0.908	0.585	0.683	0.613–0.753
Hsp90B1 (pg/mL)	730.630	0.462	0.762	0.700	0.674	0.603–0.744
OPTN (pg/mL)	511.480	0.408	0.569	0.838	0.702	0.637–0.767
Combined prediction	0.467	0.669	0.869	0.800	0.894	0.855–0.933

## 4 Discussion

Coronary artery calcification (CAC) is a pathophysiological vascular mineralization process regulated by multiple signaling pathways, with the core mechanism being the transdifferentiation of vascular smooth muscle cells (VSMCs) toward an osteogenic/chondrogenic phenotype (6). This study employed DIA quantitative proteomics to systematically characterize serum protein expression profiles in CAC patients vs. non-calcified individuals, identifying 39 significantly differentially expressed proteins. Although PCA analysis of the cohorts revealed some overlap and only moderate variance explained by disease status—a common phenomenon in serum proteomics due to high inter-individual heterogeneity—subsequent independent ELISA validation and coefficient of variation analysis in 260 individuals confirmed the robustness of our candidate biomarkers. Based on differential expression significance and functional enrichment analysis, SMOC1 (upregulated), HSP90B1 (downregulated), and OPTN (downregulated) were selected as core candidate proteins. Independent large-scale ELISA validation confirmed that the expression trends of these three proteins were fully consistent with the proteomics findings. GO enrichment analysis revealed significant enrichment of differentially expressed proteins in functional categories including “extracellular matrix,” “calcium ion binding,” “protein folding,” “unfolded protein binding,” “apoptotic process regulation,” and “immune receptor signaling pathways.” KEGG pathway enrichment indicated significant activation of “endocrine and other factor-regulated calcium reabsorption,” “T cell receptor signaling pathway,” and “Notch signaling pathway.” These findings collectively reveal the synergistic interplay of extracellular matrix remodeling, calcium homeostasis disruption, endoplasmic reticulum stress, and immune-inflammatory responses during CAC development. They further suggest that the pathological mechanisms of CAC share molecular similarities with bone development and mineralization processes.

To ensure objectivity in the selection of candidate proteins, we ranked all 39 differentially expressed proteins based on five predefined criteria: statistical significance, biological relevance, inter-assay reproducibility, supporting literature, and assay feasibility. Although STXBP2 and USH1G demonstrated outstanding statistical metrics, they were not selected due to a lack of biological association with the core CAC pathway; conversely, SMOC1, HSP90B1, and OPTN were not only statistically significant but also directly mapped to the most highly enriched GO/KEGG terms and had documented roles in calcification-related processes. This transparent, multi-criteria screening method ensured the objectivity of the selection process. Regarding statistical rigor: (1) Incremental predictive value analysis showed that the AUC of the full model was significantly higher than that of the baseline model (0.894 vs. 0.845, *P* = 0.0013), and the point estimates of NRI and IDI indicated improved clinical significance; (2) Multicollinearity tests revealed that the VIF values for all variables were <1.2, indicating a stable and reliable model; (3) Although FDR-corrected results did not reach statistical significance due to the small sample size of the discovery cohort, independent large-scale ELISA validation provided decisive evidence for the reliability of the candidate proteins.

SMOC1 belongs to the SPARC/osteocalcin family and is a secreted extracellular matrix glycoprotein containing an EF-hand calcium-binding domain. Its functions align closely with GO enrichment terms such as “calcium ion binding” and “extracellular matrix.” As a direct target gene of Runx2, a core transcription factor in osteogenic differentiation, SMOC1 plays a crucial regulatory role in skeletal development and osteoblast differentiation (7). Studies confirm that Smoc1 gene deletion causes skeletal formation disorders, manifesting as abnormal extracellular matrix assembly and mineralization defects (8). SMOC1 knockdown significantly inhibits mesenchymal stem cell mineralization and osteogenic marker expression, while overexpression promotes osteogenic differentiation (9). Combined with the significantly activated “endocrine and other factor-regulated calcium reabsorption” pathway in KEGG enrichment analysis, this suggests SMOC1 may participate in pathological vascular wall calcification through similar mechanisms. Its specific actions may involve multiple pathways: SMOC1 can interfere with growth factor signaling, such as transforming growth factor-β and bone morphogenetic protein (10, 11). Studies indicate that in a high-calcium/phosphate microenvironment, SMOC1 promotes aortic valve interstitial cell calcification by activating the p38-mediated BMP2/Smad1/5 signaling pathway (12), while the BMP signaling pathway itself promotes aortic valve calcification in VSMCs (13). phosphate microenvironment, SMOC1 promotes aortic valve interstitial cell calcification by activating the p38-mediated BMP2/Smad1/5 signaling pathway (12), and the central role of the BMP signaling pathway in the osteogenic transdifferentiation of VSMCs has been widely confirmed. Additionally, as a basement membrane matrix protein, SMOC1 interacts with extracellular matrix components, growth factors, and cell surface receptors through its multi-module structure, thereby regulating cell adhesion, migration, and signaling pathways (13, 14), which in turn influences the interactive dialogue between VSMCs and the local microenvironment. Furthermore, SMOC1 may indirectly participate in inflammatory responses by influencing platelet activity. Studies indicate SMOC1 expression in platelets enhances thrombin activity (15), and mean platelet volume has been established as an independent predictor of CAC severity (16), suggesting platelet-mediated inflammatory mechanisms may be SMOC1-related.

HSP90B1 is a core molecular chaperone protein within the heat shock protein 90 family, localized to the endoplasmic reticulum, playing a crucial role in protein folding, quality control, and maintaining endoplasmic reticulum homeostasis (17). In this study, HSP90B1 was significantly downregulated, and GO enrichment analysis revealed significant enrichment in the entries “protein folding” and “binding to unfolded proteins,” suggesting that endoplasmic reticulum stress and protein homeostasis imbalance may be involved in CAC pathogenesis. Potential mechanisms by which HSP90B1 downregulation promotes CAC include: as a key chaperone assisting the correct folding of newly synthesized proteins in the ER, reduced HSP90B1 expression impairs protein folding capacity, leading to accumulation of misfolded proteins and subsequent activation of UPR signaling pathways mediated by IRE1, ATF6, and PERK (18, 19). Excessive UPR induces VSMC apoptosis, where apoptotic bodies serve as nucleation sites for calcium salt deposition, promoting calcification. Concurrently, UPR directly stimulates VSMC osteogenic differentiation by activating osteogenic transcription factors such as RUNX2 (20, 21). Conversely, HSP90B1, as the primary calcium-binding protein in the ER, possesses multiple low-affinity, high-capacity calcium-binding sites (22). Its downregulation reduces ER calcium reserves, elevates cytoplasmic calcium ion concentrations, and subsequently activates calcium-dependent signaling pathways such as calmodulin-dependent protein kinase and NF-κB, thereby promoting VSMC osteogenic differentiation and inflammatory responses (20). The significant enrichment of the KEGG pathway “Calcium reabsorption regulated by endocrine and other factors” strongly correlates with the calcium-binding function of HSP90B1. Furthermore, as a key chaperone protein for Toll-like receptors (TLRs), HSP90B1 participates in TLR folding and transport (23, 24). Its downregulation causes TLR retention in the endoplasmic reticulum, impairing pathogen recognition and immune response capabilities (25), thereby exacerbating the inflammatory microenvironment. Activation of the TLR signaling pathway induces the release of proinflammatory factors, promoting osteogenic transformation of VSMCs (26). As an immunoregulatory factor itself, HSP90B1 downregulation may further amplify inflammatory responses. The GO enrichment of “T cell receptor signaling pathway” and “apoptotic process regulation” aligns highly with HSP90B1's immune functions.

OPTN is a multifunctional autophagy receptor protein involved in selective autophagy, vesicle transport, inflammatory signaling, and endoplasmic reticulum stress regulation (27, 28). This study revealed that OPTN is significantly downregulated in CAC, with GO enrichment analysis showing significant enrichment in the “apoptotic process regulation” and “T cell receptor signaling pathway” entries. Autophagy is a key mechanism for maintaining intracellular homeostasis. As an autophagy receptor, OPTN specifically recognizes ubiquitinated substrates and delivers them to autophagolysosomes for degradation (29). In VSMCs, impaired autophagy has been shown to promote cellular senescence, apoptosis, and osteoblast-like transformation, thereby accelerating vascular calcification (30). OPTN downregulation may disrupt the autophagic flux, leading to the accumulation of damaged mitochondria and misfolded proteins, thereby inducing mitochondrial dysfunction, oxidative stress, and apoptosis. Studies indicate that OPTN-deficient cells exhibit heightened sensitivity to endoplasmic reticulum stress inducers and increased cathepsin-3 activation (31), suggesting that OPTN deficiency may exacerbate cellular damage by impairing autophagic clearance capacity. OPTN also directly participates in endoplasmic reticulum stress regulation by interacting with the stress sensor IRE1α to modulate its protein levels (32), thereby maintaining ER homeostasis. ER stress is a key trigger for vascular calcification; excessive UPR activation induces VSMC apoptosis and osteogenic differentiation (33), and OPTN downregulation may lead to overactivation of the UPR signaling pathway (34). Regarding inflammatory regulation, OPTN suppresses basal and TNFα-induced NF-κB activation (35) and downregulates T cell receptor-induced NF-κB activation and TNF-α production (36). NF-κB activation and TNF-α production are significantly enhanced in OPTN knockout cells, and inflammatory responses play a critical role in vascular calcification (37). GO enrichment analysis reveals strong alignment between “T cell receptor signaling pathway” and “apoptotic process regulation” enrichment and OPTN's immune regulatory functions. OPTN also participates in vesicle transport (38) and oxidative stress response regulation (39), and its downregulation may further disrupt cellular homeostasis.

Although numerous previous studies on serum proteomics in coronary artery calcification have provided important methodological and mechanistic references for this study, the present study still exhibits significant differences in study design, target identification, mechanistic dimensions, and clinical models, thereby offering unique added value. Zhang et al. employed a DIA strategy to analyze serum samples from patients with severe CAC and controls, identifying differentially expressed proteins such as complement C5, FGG, and PKM2. These proteins were primarily enriched in the complement-coagulation cascade, platelet activation, and glycolysis pathways, highlighting inflammation and metabolic reprogramming (40); Royer et al. (41) constructed a predictive model for subclinical CAC based on plasma proteomics in a community-based cohort, emphasizing the predictive efficacy of proteins beyond traditional risk factors; Mancio et al. (42) focused on epicardial fat and found that the Annexin A2/Fetuin-A axis is associated with severe coronary artery calcification, suggesting a regulatory role of the local microenvironment; Meanwhile, El-Sabawi et al. (43) conducted a large-scale proteomics and genetic integration analysis, systematically identifying targets such as GDF-15, MMPs, the Notch pathway, PCSK9, and APOC1. They linked the proteome with genetic susceptibility and coronary-specific transcriptomics, elucidating the synergistic effects of inflammation, matrix remodeling, calcification signaling, and metabolic dysregulation. While the aforementioned studies focused on severe calcification, community-based prevention cohorts, or the integration of local tissue and genetic data, none achieved a complete translational pathway that simultaneously incorporates unbiased high-throughput DIA screening, large-sample independent serum validation, and the construction of a multi-molecular predictive model. Compared with previous studies, the innovation of this study lies in three aspects: First, while previous CAC proteomics studies have primarily focused on proteins related to inflammation, coagulation, metabolism, and matrix remodeling, this study is the first to discover that the three serum proteins SMOC1, HSP90B1, and OPTN—three serum proteins—to be stably associated with CAC. These proteins target calcium binding and osteogenic signaling, endoplasmic reticulum stress and protein homeostasis, and autophagy and inflammatory regulation, respectively, thereby supplementing the existing mechanistic landscape with a new dimension of “calcium metabolism–organelle stress–cellular quality control”; Second, while most studies stop at biomarker screening and single-molecule validation, this study integrates protein biomarkers with clinical indicators to construct a visualized nomogram. ROC curves, calibration curves, and DCA have confirmed its strong predictive ability and clinical net benefit, providing a tool that can be directly used for risk stratification; Third, while existing protein biomarkers largely overlap with traditional inflammatory or metabolic pathways, the SMOC1 identified in this study—as a Runx2 downstream calcium-binding protein—directly participates in vascular mineralization, and HSP90B1 and OPTN regulate the osteogenic transdifferentiation of vascular smooth muscle cells via the endoplasmic reticulum stress–autophag *y* axis. These represent non-classical, mechanism-oriented candidate targets that can provide new directions for mechanistic research and intervention development. Concurrently, this study also validated the independent roles of traditional factors such as age, uric acid, alkaline phosphatase, and fasting blood glucose, achieving complementary advantages between new protein targets and classic clinical indicators, thereby enhancing the model's stability and interpretability. In summary, building upon previous proteomics paradigms, this study has achieved progressive innovation through the discovery of new targets, deeper elucidation of mechanisms, and the implementation of clinical models, providing a candidate combination for serum-based CAC risk assessment that is more specific and mechanistically relevant. Therefore, although the technical workflow is standard, the specific biomarker combination, the biological pathways it suggests, and the validated predictive model represent meaningful innovation.

In summary, this study reports for the first time the differential expression of SMOC1, HSP90B1, and OPTN in serum samples from patients with coronary artery calcification (CAC). These findings were validated through independent large-scale ELISA assays, with expression trends fully consistent with proteomics discoveries. Functional enrichment analysis revealed that the differentially expressed proteins were significantly enriched in biological processes and signaling pathways, including extracellular matrix remodeling, endoplasmic reticulum stress, autophagy, and immune-inflammatory regulation. This aligns closely with the known functions of SMOC1, HSP90B1, and OPTN, suggesting that these proteins may participate in the development and progression of CAC through the aforementioned mechanisms. To date, no literature has directly reported the relationship between SMOC1, HSP90B1, and OPTN with CAC. Their specific mechanisms in vascular calcification remain unexplored. The proposed mechanism hypothesis based on proteomics findings and bioinformatics analysis requires further validation through subsequent basic experiments.

The innovation of this study lies in the first-ever integration of novel serum biomarkers identified through DIA quantitative proteomics screening with traditional clinical indicators to construct a CAC risk prediction nomogram model. Multivariate logistic regression analysis revealed that age, serum uric acid, alkaline phosphatase, fasting blood glucose, SMOC1, HSP90B1, and OPTN are independent risk factors for CAC. The nomogram model constructed based on these indicators demonstrated excellent discriminatory ability (AUC = 0.894, 95% CI: 0.855–0.933). Calibration curves showed high concordance between predicted and observed probabilities, while decision curve analysis indicated positive clinical net benefit within the 0.1–0.8 probability threshold range. This nomogram integrates traditional risk factors with novel serum protein biomarkers. All indicators are obtained through serum testing, offering advantages such as minimal invasiveness, high accessibility, and ease of operation. It provides a practical tool for individualized risk assessment and clinical decision-making regarding CAC, demonstrating promising prospects for clinical translation. Our blood-based model is not intended to replace the current gold standard of CCTA/CACS, but rather to serve as an initial triage tool within a “screening-diagnosis” stepwise strategy. This model offers the following advantages: (1) It can be used in populations unsuitable for CCTA (e.g., those with contrast agent allergy, renal insufficiency, or pregnancy) and in resource-limited settings; (2) The three protein biomarkers are dynamic and can be used to monitor treatment response, which is difficult to achieve with CACS due to radiation exposure; (3) Receiver operating characteristic (ROC) curve analysis (Figure 16) demonstrates a net positive benefit within a broad threshold range of 0.1–0.8, supporting its use in clinical decision-making. The model's potential value is greatest in the intermediate-risk population, as traditional risk scores (such as Framingham) have poor discriminatory power in this group. We recommend the following workflow: (1) Use the blood-based model for initial screening in the general population or in individuals with unclear traditional risk indicators; (2) Refer high-risk individuals (above a predetermined threshold) for CCTA/CACS confirmation; (3) Low-risk individuals may undergo conservative lifestyle intervention and follow-up. This strategy balances efficiency, safety, and cost-effectiveness. However, we acknowledge that prior to clinical implementation, the model requires: (a) multicenter external validation to recalibrate the optimal probability threshold; (b) development of a standardized ELISA kit with a unified calibration standard; (c) a formal health economic cost-effectiveness analysis.

Several limitations of this study warrant clarification. First, this study was designed as a single-center trial, with all participants recruited from the Inner Mongolia region of China (a Han Chinese population in northern China). The lack of an independent, multicenter external validation cohort is a major limitation of this study, which restricts the generalizability of the findings and the predictive model. Therefore, the applicability of the current model is limited to populations with similar epidemiological characteristics. Second, during the discovery phase, we employed a sample pooling strategy to balance detection sensitivity and cost given the limited sample size; however, this method cannot assess the degree of inter-individual variation in protein expression. We partially addressed this limitation by validating the model in a subsequent independent large-sample ELISA dataset (*n* = 260). Third, although the validation cohort is independent of the discovery cohort, both originated from the same center, and the predictive model was validated only internally. The model's generalizability and robustness require further confirmation using multicenter external data. Fourth, the cross-sectional design of this study cannot establish a causal relationship between changes in SMOC1, HSP90B1, and OPTN expression and the development of CAC; their specific molecular mechanisms in vascular calcification remain to be elucidated through cellular and animal experiments. Based on the above limitations, we propose clear directions for future research. Regarding external validation, we recommend conducting a multicenter study involving ≥2 independent medical centers, adopting uniform inclusion/exclusion criteria, a standardized CCTA protocol, and an ELISA testing procedure, with a planned sample size of ≥500 cases. The validation objectives include: (1) confirming the expression trends of SMOC1, HSP90B1, and OPTN; (2) evaluating the discriminatory power (AUC), calibration, and clinical net benefit (DCA) of the receiver operating characteristic (ROC) curve in an external population; (3) assessing the model's performance across different calcification severity strata and demographic subgroups. This external validation is a prerequisite for any future clinical application. Regarding mechanistic studies, basic experiments should further elucidate the specific roles of SMOC1, HSP90B1, and OPTN in the osteogenic differentiation of vascular smooth muscle cells and calcium salt deposition. The aforementioned studies will provide new strategies and targets for the early identification, risk stratification, and precision prevention and treatment of CAC.

## Data Availability

The mass spectrometry proteomics data presented in the study are deposited in the ProteomeXchange Consortium (http://proteomecentral.proteomexchange.org) via the iProX partner repository, accession number PXD075378.
