# Supplementary material for: Screening of serum biomarkers for coronary artery calcification using DIA quantitative proteomics and construction of a regression model
Source: Front Cardiovasc Med. 2026 Jun 1;13:1824102. doi: 10.3389/fcvm.2026.1824102 (PMC13265296; doi:10.3389/fcvm.2026.1824102)
Supplement: Supplementary file 1 [file Datasheet1.docx]

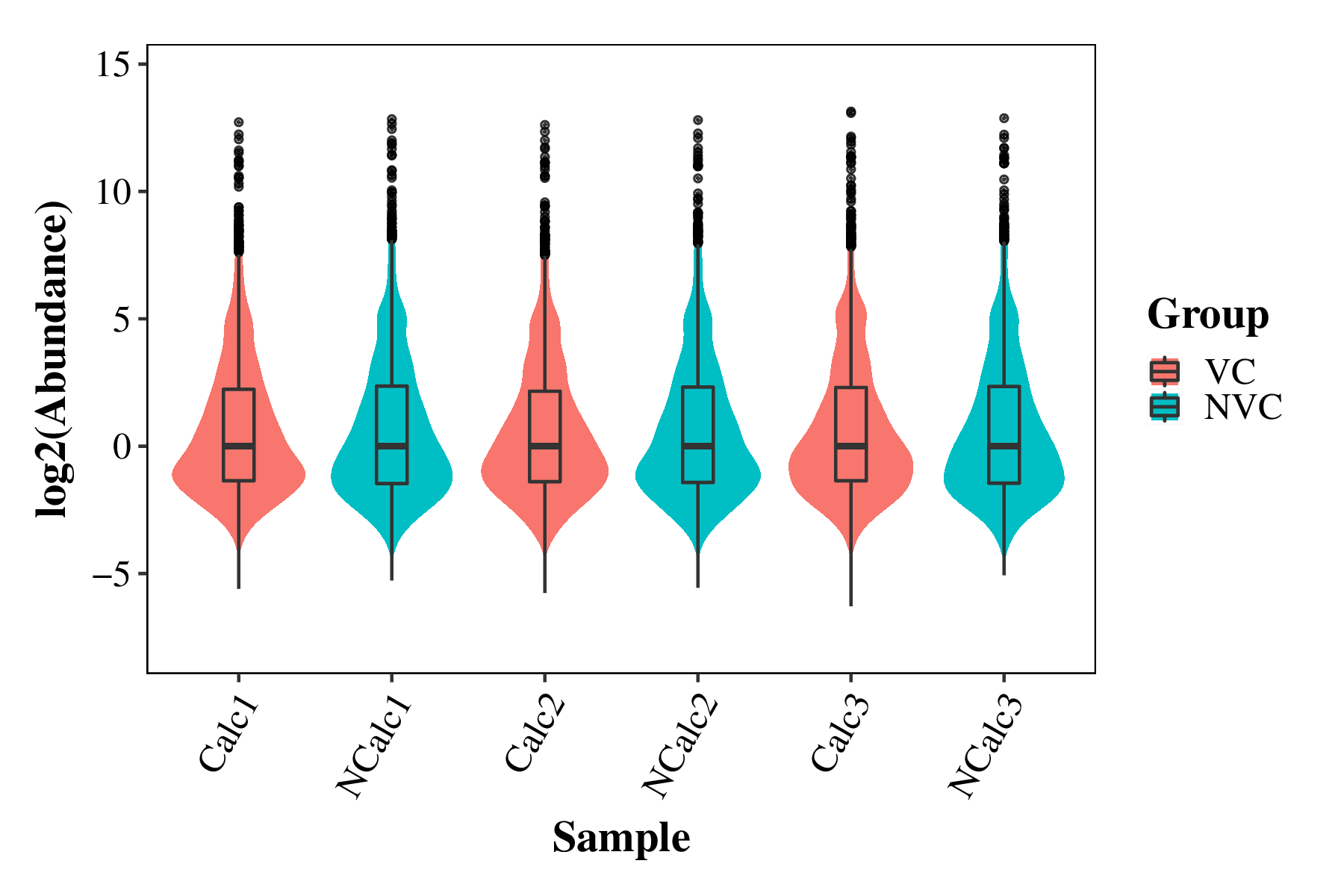


Figure S1 Distribution of Protein Abundance Values in Serum Samples Across Groups


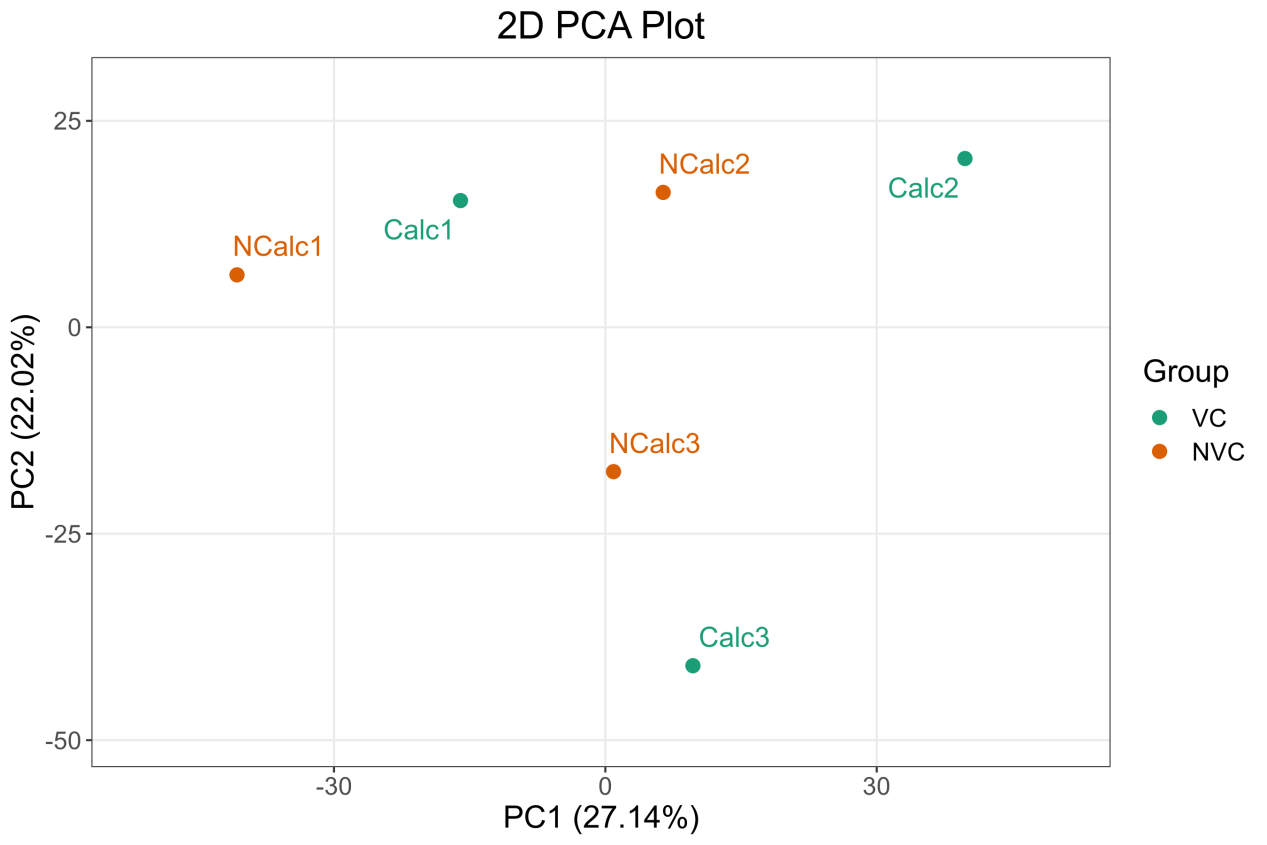


Figure S2: Two-dimensional score plot of principal component analysis (PCA) for serum proteomics


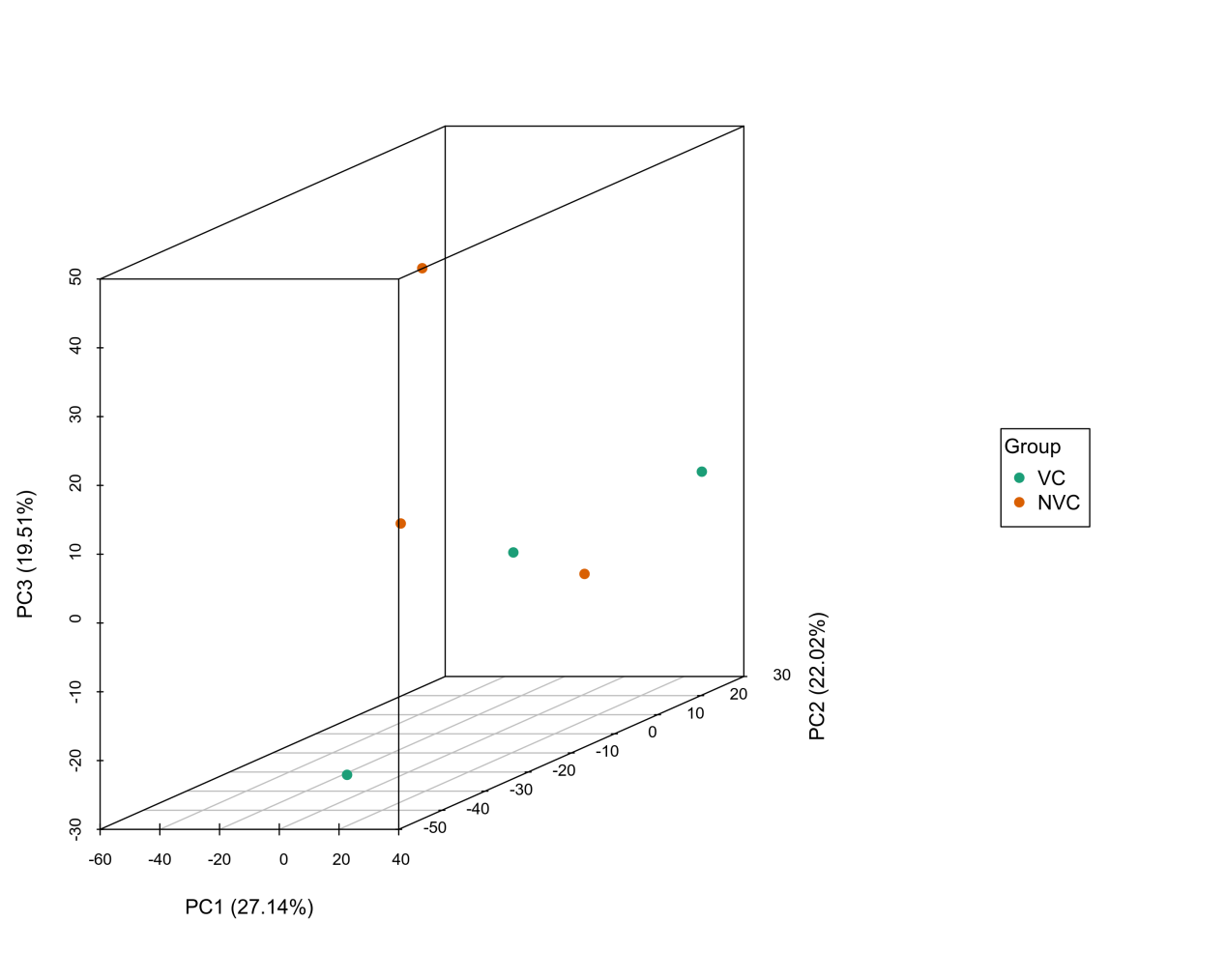


Figure S3: Three-dimensional distribution plot of principal component analysis (PCA) for serum proteomics


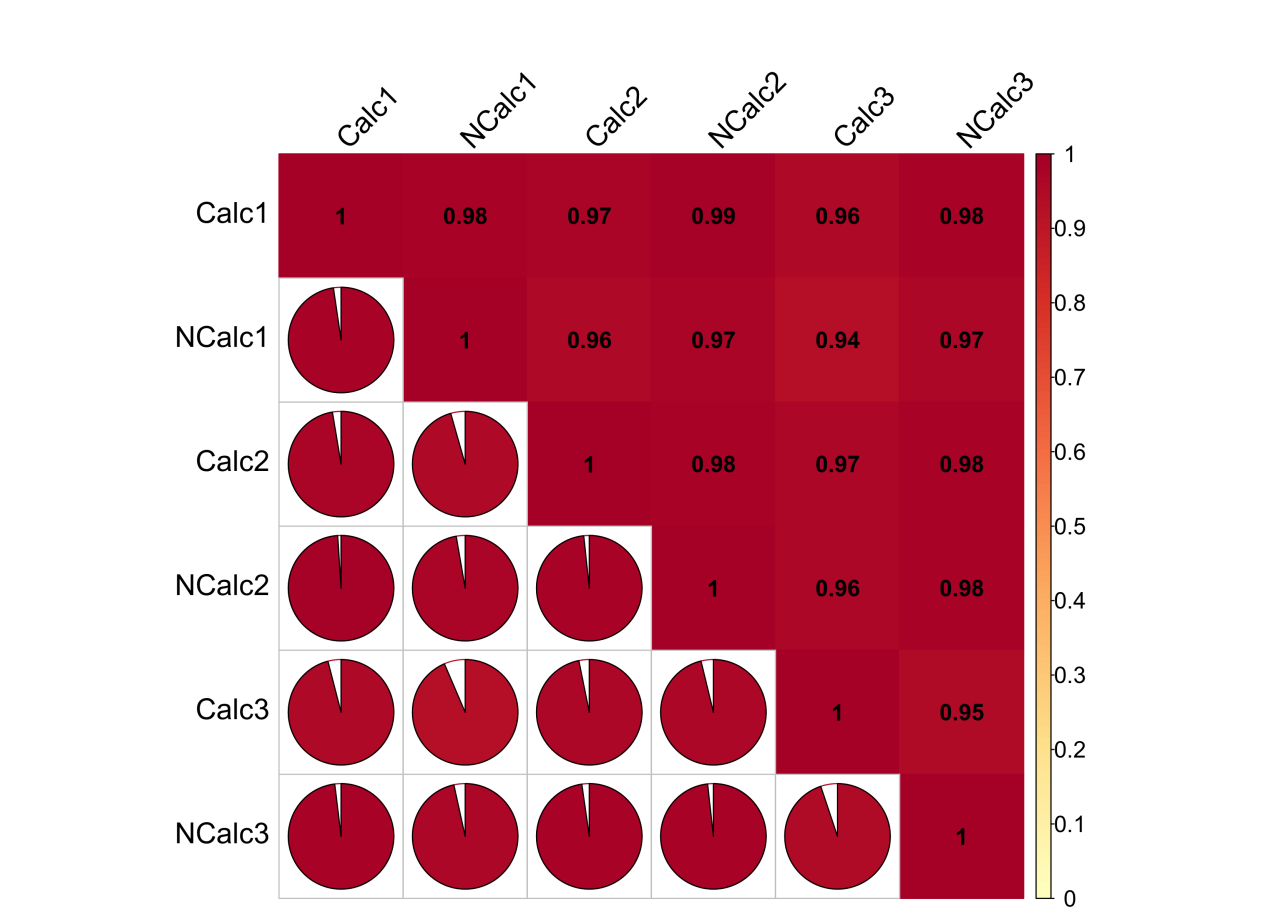


Figure S4: Heatmap of Correlation Among Serum Samples Across Groups


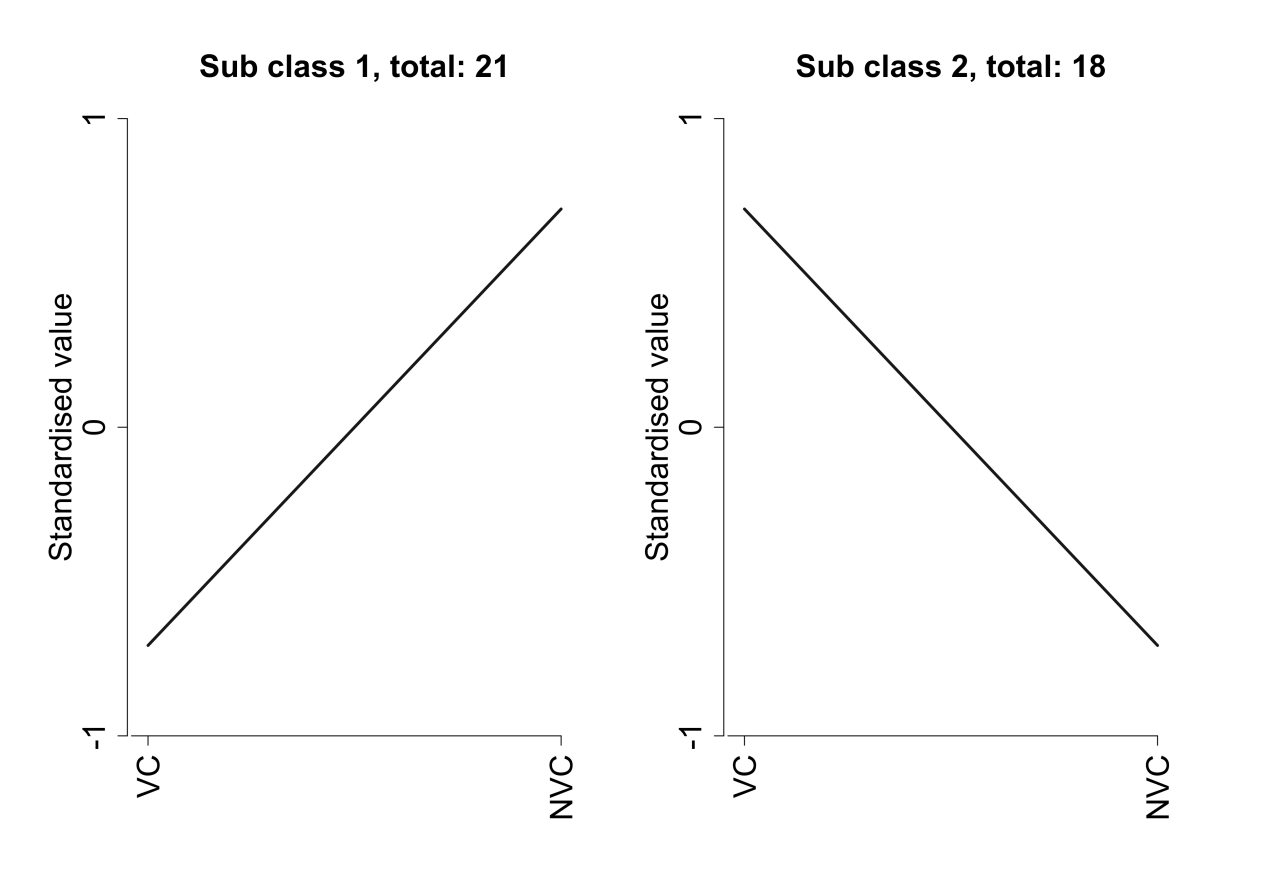


Figure S5: K-means Clustering Analysis of Differentially Expressed Proteins


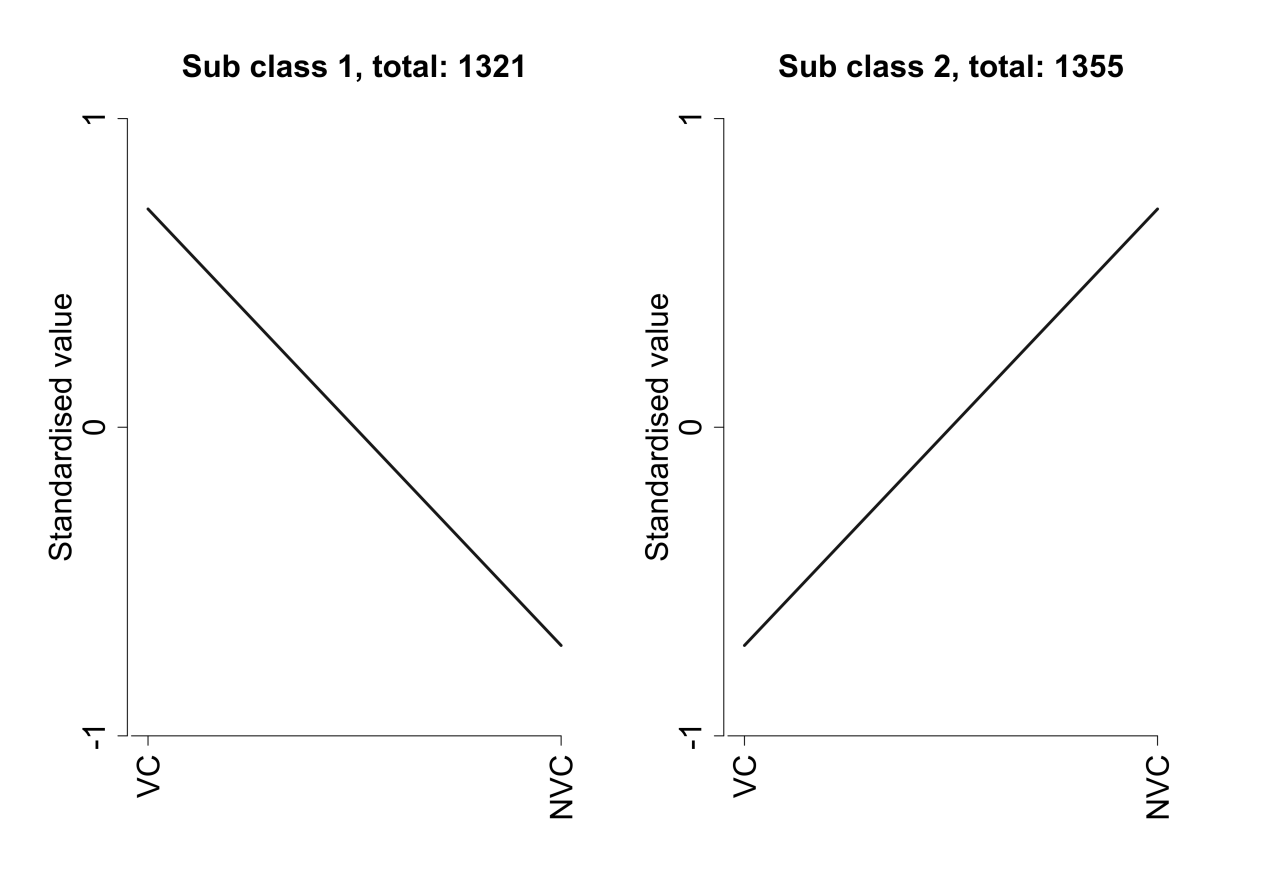


Figure S6: K-means Clustering Analysis Diagram for All Quantitative Proteins


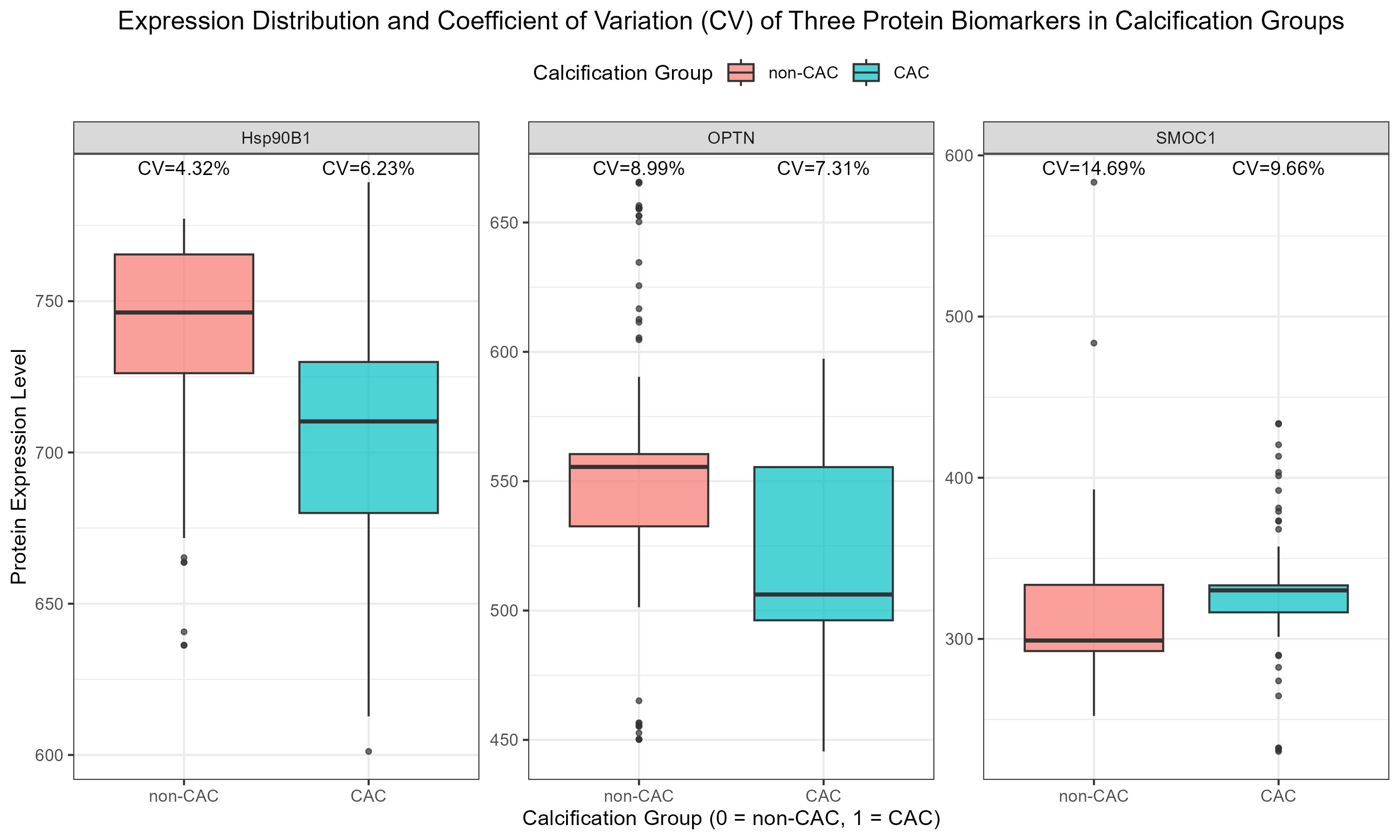


Figure S7 Expression distribution and coefficient of variation (CV) of three candidate proteins in the discovery cohort


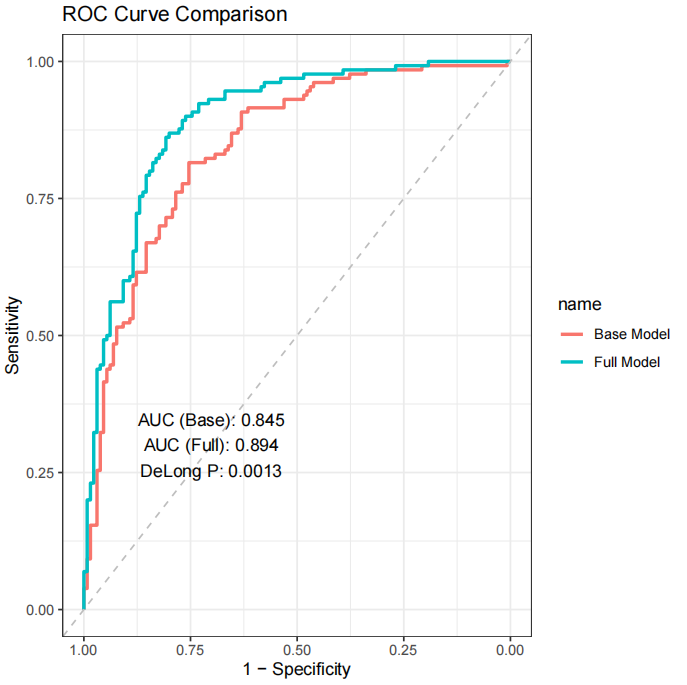


Figure S8. Receiver operating characteristic (ROC) curves comparing the predictive performance of the base model and full model for coronary artery calcification.


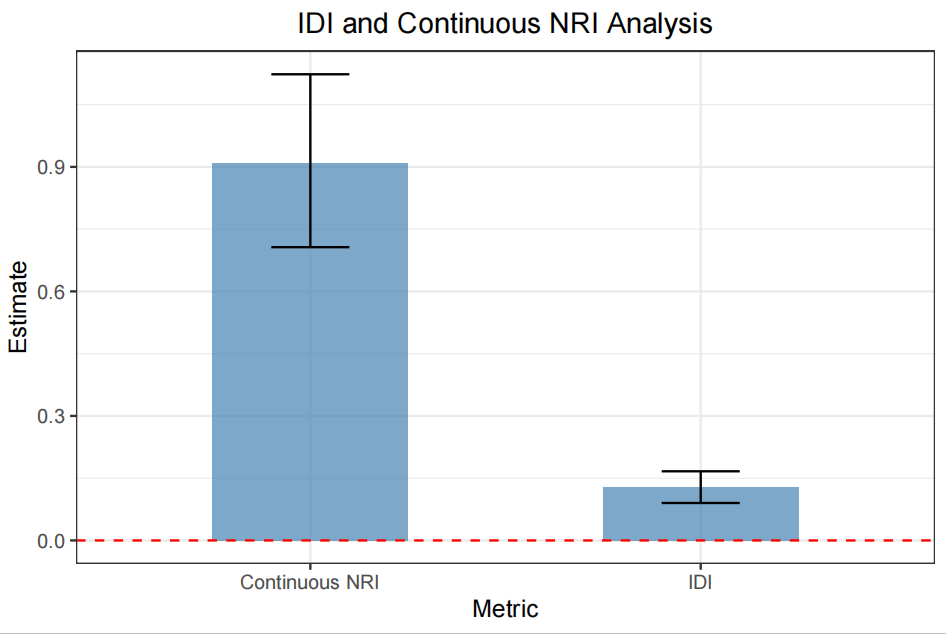


Figure S9. Net reclassification improvement (NRI) and integrated discrimination improvement (IDI) for the full model versus the base model.


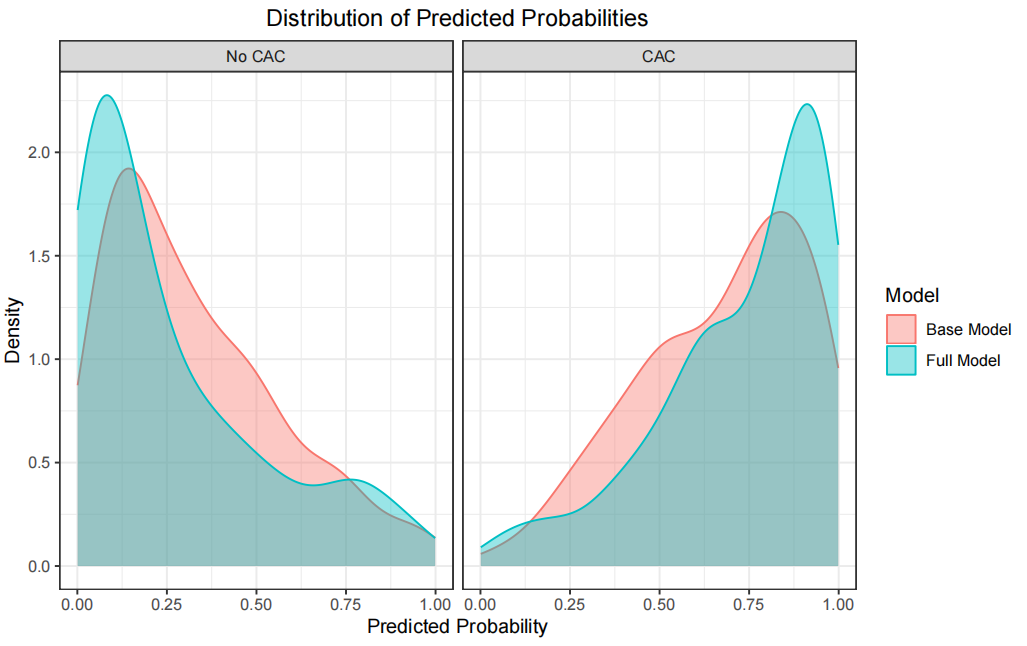


Figure S10. Distribution of predicted probabilities from the base model and full model in CAC and non-CAC groups.

Supplementary Table S1. Multicollinearity assessment of predictors in the base model and full model using variance inflation factor (VIF)

| Model | Variable | VIF |
| --- | --- | --- |
| Base Model (Clinical Indicators) | age | 1.091 |
| Base Model (Clinical Indicators) | BUA | 1.033 |
| Base Model (Clinical Indicators) | ALP | 1.052 |
| Base Model (Clinical Indicators) | FBG | 1.115 |
| Full Model (Clinical + Proteins) | age | 1.072 |
| Full Model (Clinical + Proteins) | BUA | 1.037 |
| Full Model (Clinical + Proteins) | ALP | 1.051 |
| Full Model (Clinical + Proteins) | FBG | 1.14 |
| Full Model (Clinical + Proteins) | SMOC1 | 1.085 |
| Full Model (Clinical + Proteins) | Hsp90B1 | 1.028 |
| Full Model (Clinical + Proteins) | OPTN | 1.093 |

Supplementary Table S2. List of differentially expressed proteins between the coronary artery calcification group and non-calcification control group with false discovery rate (FDR)-adjusted P-values.

| Accession | Gene | FC | P-value | Regulation | FDR |
| --- | --- | --- | --- | --- | --- |
| P17252 | PRKCA | 2.05489398 | 0.000675239 | up | 0.710088109 |
| Q9UQ35 | SRRM2 | 1.504920599 | 0.005619739 | up | 0.986094939 |
| Q8TCD5 | NT5C | 1.552215864 | 0.013184806 | up | 0.986094939 |
| O75487 | GPC4 | 1.937706494 | 0.013316717 | up | 0.986094939 |
| P62241 | RPS8 | 1.982530867 | 0.013381255 | up | 0.986094939 |
| Q8N2S1 | LTBP4 | 1.932997137 | 0.017821308 | up | 0.986094939 |
| P13010 | XRCC5 | 2.134981721 | 0.020055773 | up | 0.986094939 |
| D6RBQ9 | HNRNPD | 1.679102568 | 0.020238896 | up | 0.986094939 |
| P52790 | HK3 | 1.911492645 | 0.020919192 | up | 0.986094939 |
| P31939 | ATIC | 2.092901586 | 0.024706548 | up | 0.986094939 |
| A0A0C4DH34 | IGHV4-28 | 1.698334089 | 0.027182897 | up | 0.986094939 |
| Q9H4F8 | SMOC1 | 2.786170463 | 0.029640303 | up | 0.986094939 |
| P36578 | RPL4 | 1.550350723 | 0.036282877 | up | 0.986094939 |
| A0AAG2TF08 | CANX | 1.506803966 | 0.037224876 | up | 0.986094939 |
| Q15833 | STXBP2 | 3.860368808 | 0.043502717 | up | 0.986094939 |
| Q9Y315 | DERA | 2.61691839 | 0.04370748 | up | 0.986094939 |
| Q96RF0 | SNX18 | 1.818647296 | 0.045863443 | up | 0.986094939 |
| P29350 | PTPN6 | 1.554238337 | 0.04781798 | up | 0.986094939 |
| P30740 | SERPIN | 0.627467112 | 0.000356601 | down | 0.710088109 |
| Q9NPA2 | MMP25 | 0.641705509 | 0.001098015 | down | 0.734571903 |
| Q9Y6R7 | FCGBP | 0.425251713 | 0.006504694 | down | 0.986094939 |
| J3KSN5 | USH1G | 0.391267176 | 0.008503667 | down | 0.986094939 |
| Q9H3G5 | CPVL | 0.463004698 | 0.01143002 | down | 0.986094939 |
| Q9NRR1 | CYTL1 | 0.489202925 | 0.012423649 | down | 0.986094939 |
| P43251 | BTD | 0.64644192 | 0.01426334 | down | 0.986094939 |
| Q15166 | PON3 | 0.543418135 | 0.014356101 | down | 0.986094939 |
| Q8NEJ9 | NGDN | 0.438083612 | 0.015273007 | down | 0.986094939 |
| Q15848 | ADIPOQ | 0.509027766 | 0.0231487 | down | 0.986094939 |
| Q13200 | PSMD2 | 0.647639374 | 0.023568757 | down | 0.986094939 |
| Q96CV9 | OPTN | 0.415523192 | 0.029592589 | down | 0.986094939 |
| A0A8V8TNU2 | WDR1 | 0.53589367 | 0.030413633 | down | 0.986094939 |
| Q9NY33 | DPP3 | 0.600977212 | 0.035671742 | down | 0.986094939 |
| P28300 | LOX | 0.376211602 | 0.036667755 | down | 0.986094939 |
| Q99685 | MGLL | 0.433260279 | 0.038914328 | down | 0.986094939 |
| P42680 | TEC | 0.519382135 | 0.041853216 | down | 0.986094939 |
| Q8NES3 | LFNG | 0.645894824 | 0.042533724 | down | 0.986094939 |
| A6NMY6 | ANXA2P2 | 0.54015034 | 0.045223555 | down | 0.986094939 |
| Q9NVJ2 | ARL8B | 0.645711447 | 0.047331537 | down | 0.986094939 |
| A0A7P0TAY2 | HSP90B1 | 0.617428484 | 0.049683883 | down | 0.986094939 |

Table S3. Distribution of Coronary Artery Calcium Score (CACS) in the study population

| Group | N | Mean ± SD | Median [Q1, Q3] | Range |
| --- | --- | --- | --- | --- |
| Non-CAC | 130 | 0 ± 0 | 0 [0, 0] | 0 - 0 |
| CAC | 130 | 301.9 ± 389.5 | 86.6 [70.6, 319.6] | 49.43 - 1518.89 |


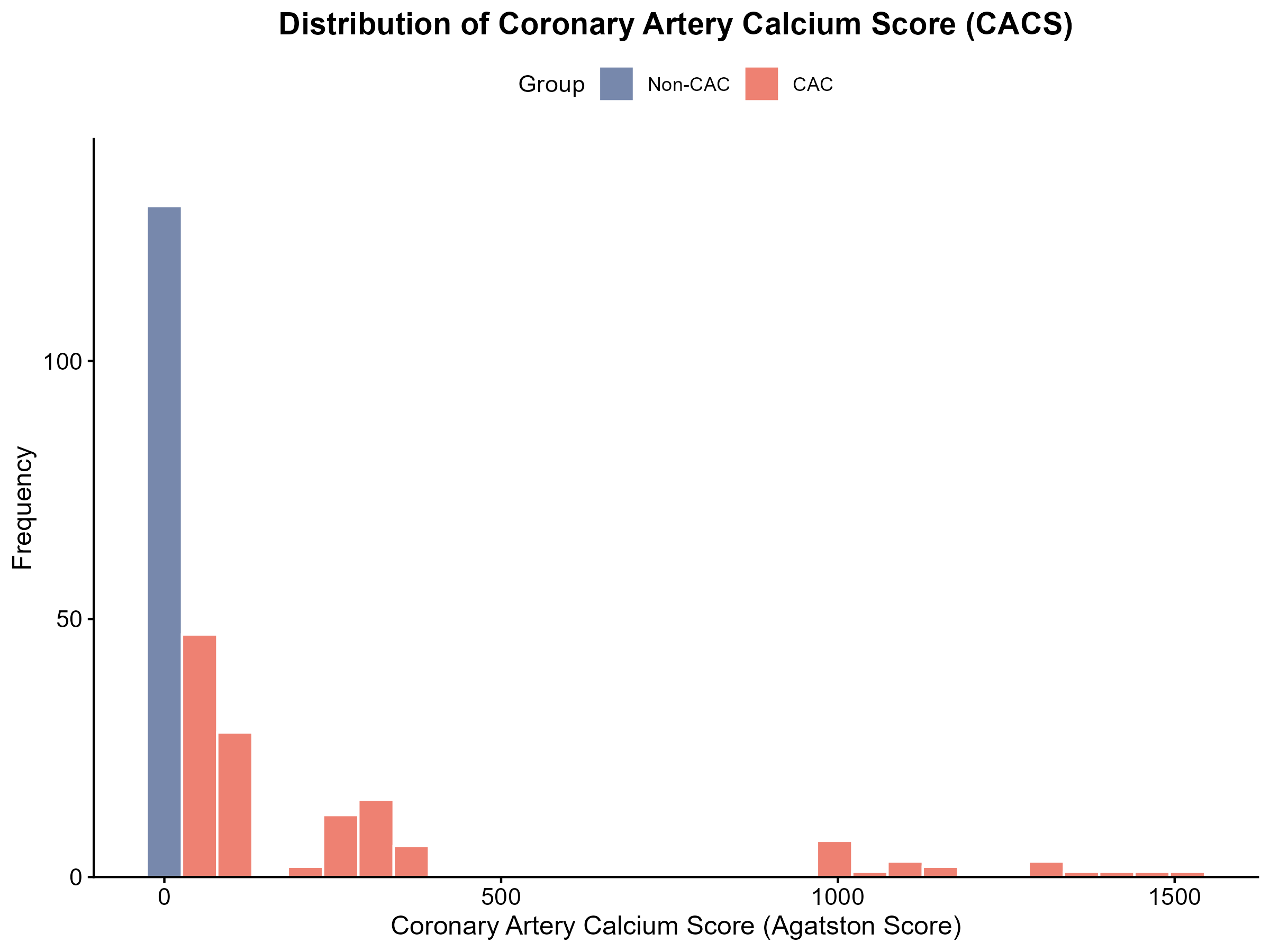


Figure S11 Distribution of Coronary Artery Calcium Score (CACS, Agatston score) in the study population.

The blue bars represent the Non-CAC group (n = 130, all participants with a CACS of 0). The red bars represent the CAC group (n = 130), which exhibits a right-skewed distribution with a median CACS of 86.6 (interquartile range: 70.6–319.6) and a range from 49.43 to 1518.89.
